# Genetic factors underlying discordance in chromatin accessibility between monozygotic twins

**DOI:** 10.1186/gb-2014-15-5-r72

**Published:** 2014-05-29

**Authors:** Kwoneel Kim, Hyo-Jeong Ban, Jungmin Seo, Kibaick Lee, Maryam Yavartanoo, Sang Cheol Kim, Kiejung Park, Seong Beom Cho, Jung Kyoon Choi

**Affiliations:** 1 Department of Bio and Brain Engineering, KAIST, Daejeon 305-701, Republic of Korea; 2 Department of Biomedical Informatics, Center for Genome Science, National Institute of Health, KCDC, Choongchung-Buk-do 363-951, Republic of Korea; 3 Division of Molecular and Life Sciences, Hanyang University, Ansan, Gyeonggi-do 425-791, Republic of Korea; 4 Research Institute of Bioinformatics, Omicsis, Inc., Daejeon 305-333, Republic of Korea; 5 Samsung Genome Institute, Samsung Medical Center, Seoul 135-710, Republic of Korea

## Abstract

**Background:**

Open chromatin is implicated in regulatory processes; thus, variations in chromatin structure may contribute to variations in gene expression and other phenotypes. In this work, we perform targeted deep sequencing for open chromatin, and array-based genotyping across the genomes of 72 monozygotic twins to identify genetic factors regulating co-twin discordance in chromatin accessibility.

**Results:**

We show that somatic mutations cause chromatin discordance mainly via the disruption of transcription factor binding sites. Structural changes in DNA due to C:G to A:T transversions are under purifying selection due to a strong impact on chromatin accessibility. We show that CpGs whose methylation is specifically regulated during cellular differentiation appear to be protected from high mutation rates of 5′-methylcytosines, suggesting that the spectrum of CpG variations may be shaped fully at the developmental level but not through natural selection. Based on the association mapping of within-pair chromatin differences, we search for cases in which twin siblings with a particular genotype had chromatin discordance at the relevant locus. We identify 1,325 chromatin sites that are differentially accessible, depending on the genotype of a nearby locus, suggesting that epigenetic differences can control regulatory variations via interactions with genetic factors. Poised promoters present high levels of chromatin discordance in association with either somatic mutations or genetic-epigenetic interactions.

**Conclusion:**

Our observations illustrate how somatic mutations and genetic polymorphisms may contribute to regulatory, and ultimately phenotypic, discordance.

## Background

Open chromatin provides access for a wide spectrum of DNA binding proteins to regulate transcription, DNA repair, recombination, replication, and so on. As such, open chromatin profiling has been used to identify the genomic locations of various regulatory regions, including promoters, enhancers, insulators, silencers, replication origins, and recombination hotspots [1-4]. The binding sites for transcription factors (TFs) have been extensively profiled based on the distribution of sequencing tags derived from DNase I hypersensitive sites [5,6]. The FAIRE (formaldehyde-assisted isolation of regulatory elements) technique has also been used to capture open chromatin regions in the genome [2,7-11].

Chromatin accessibility has been a focal point in the studies exploring the intersection of genetics and epigenetics. Meanwhile, coupling between chromatin accessibility and underlying genetic polymorphisms renders chromatin status a heritable feature [12]. In a recent study [13], association mapping was used to understand the genetic basis of chromatin regulation, and in our previous work [11], we made a similar attempt based on the genetic linkage of FAIRE signals. It was also demonstrated that disease-associated regulatory variations could be mapped to FAIRE regions [9] or DNase I hypersensitive sites [14]. Furthermore, a method based on FAIRE DNA genotyping for the systematic identification of regulatory polymorphisms associated with different phenotypes has also been introduced [10]. However, how somatic mutations are able to affect chromatin accessibility still remains to be elucidated.

While monozygotic (MZ) twins are useful for studying epigenetic differences caused by differential environmental exposure, DNA methylation is the only epigenetic mechanism that has been studied in depth with regard to MZ discordance [15-17]. A recent study [18] showed that DNA methylation could function as an intermediary of genetic factors associated with particular traits or phenotypes. However, the modulation of chromatin structure is central to epigenetic regulation, and chromatin accessibility in particular can be linked directly to transcriptional activity [1,13] through a combination of multiple epigenetic mechanisms, including DNA methylation. Yet despite the importance of open chromatin in transcriptional regulation, MZ discordance in chromatin accessibility has never been explored.

In this work, we investigated co-twin chromatin discordance and the associated genetic factors. We first sought to obtain the full spectrum of somatic and genetic single nucleotide variations that underlie open chromatin. To this end, we compared the patterns of somatic mutations (that is, within-pair sequence differences) with those of genetic polymorphisms (that is, between-pair sequence differences). In addition, we attempted to characterize genetic-epigenetic interactions by finding genetic polymorphisms that influence within-pair differences in chromatin accessibility. Thus, we performed quantitative trait loci (QTL) mapping for quantitative within-pair chromatin differences across twin pairs.

## Results and discussion

We performed high-quality, in-depth open chromatin sequencing (approximately 72×, with 92% of Q ≥30 bases; Additional file 1) for 36 pairs of MZ twins. We selected twins aged between 30 and 60 years who were discordant for immunological traits, mostly involving allergic symptoms (Additional file 2), so that more somatic differences in immune cells could be observed. Normalized chromatin accessibility was determined for the identified open chromatin regions. Chromatin accessibility showed a higher correlation between twin siblings than between unrelated individuals (Additional file 3). Our data showed good agreement with the public FAIRE and DNase I data in GM12878 lymphoblastoid cells (Additional file 4). To assess the accuracy of our sequencing-based variant detection method, we employed Affymetrix 6.0 SNP arrays to genotype peripheral blood DNA. A concordance rate of approximately 76% was observed when lower confidence variants were included to increase the overlap with the array data (see Materials and methods; Additional files 5 and 6). Somatic regulatory mutations, defined as variants that resided in open chromatin and significantly differed between twin siblings, were identified and confirmed to an unprecedented level thanks to targeted deep sequencing of open chromatin in many individuals. Meanwhile, regulatory polymorphisms (SNPs) were defined as variants that showed a minimum allele frequency of 1% among all the FAIRE reads across all samples (see Materials and methods).

**Figure 1:**
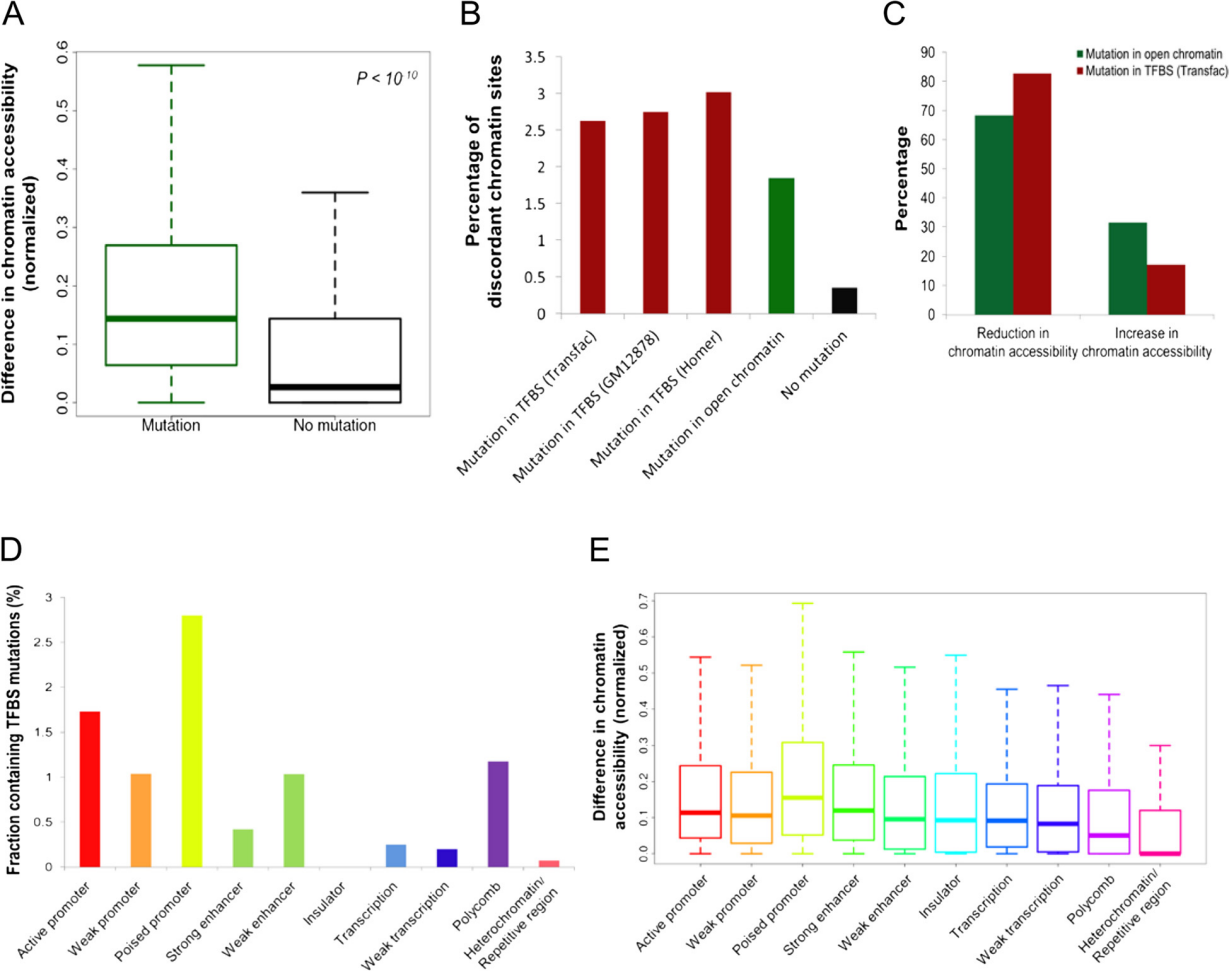
**Effect of somatic mutations on chromatin regulation. (A)** Within-pair differences in chromatin accessibility based on the presence and absence of mutations. **(B)** Correlation of chromatin discordance with the presence of mutations in open chromatin and in TFBSs as predicted based on Transfac position weight matrices, motif enrichment analysis by Homer, and ChIP-seq data in GM12878 lymphoblastoid cells. **(C)** Correlation of chromatin discordance with mutation types. **(D,E)** Within-pair differences in mutation rates **(D)** and chromatin accessibility (E) according to different regulatory states.

**Figure 2:**
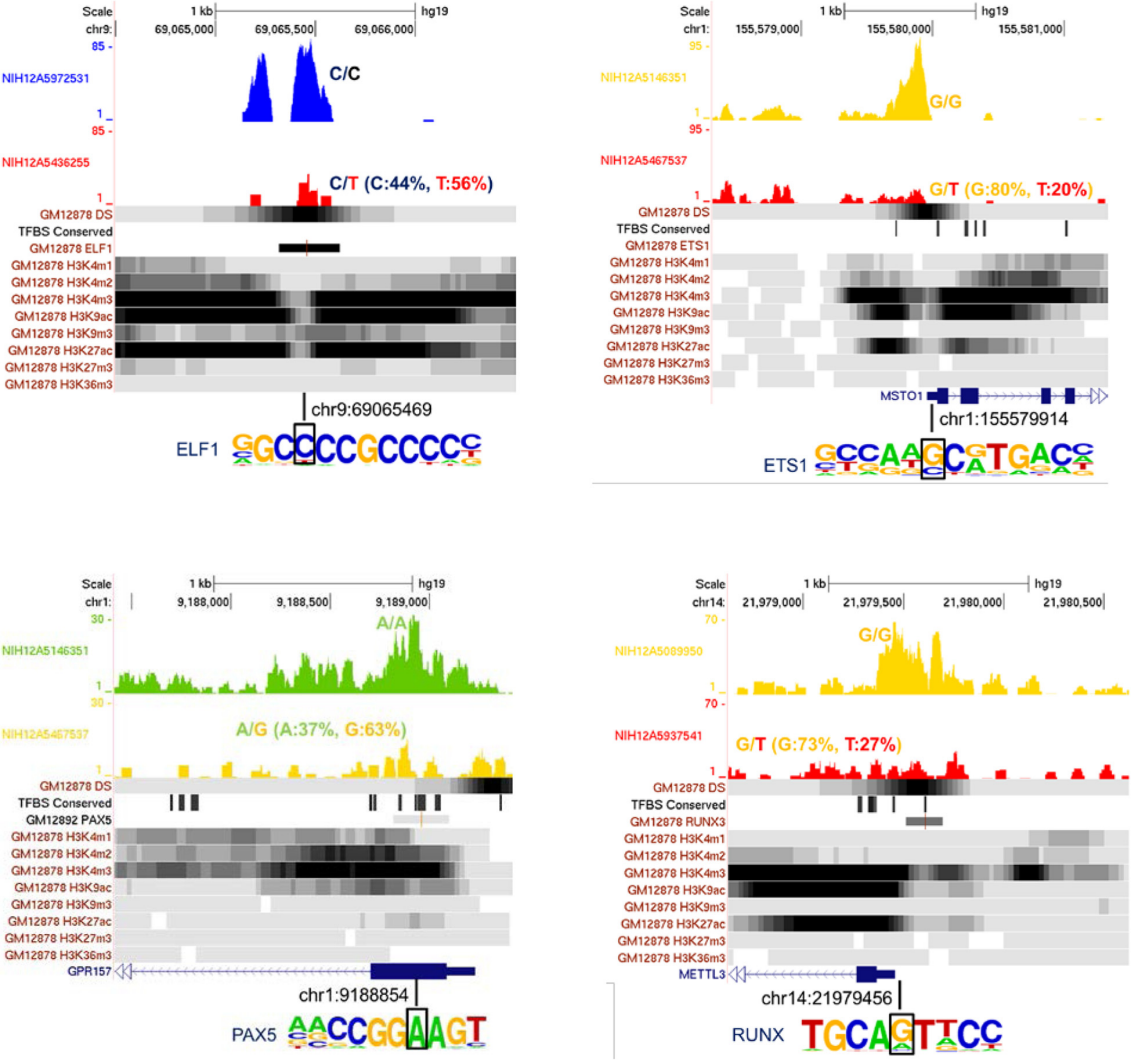
**Examples of mutations that decrease chromatin accessibility by disrupting TF recognition motifs.** Shown below the accessibility signals from the MZ twins are the DNase I clusters, TF binding signals, conserved TFBSs and histone modification patterns derived from the ENCODE data in GM12878 lymphoblastoid cells.

**Figure 3:**
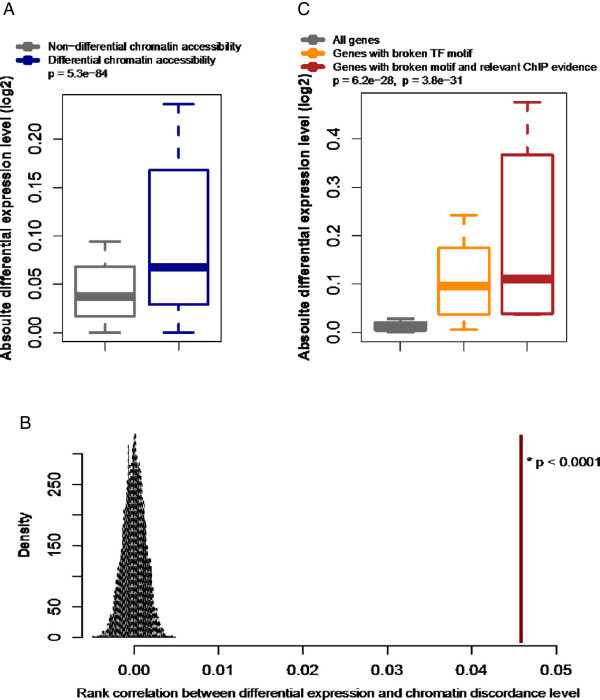
**Effect of chromatin discordance and somatic mutation on differential expression. (A)** Within-pair differential expression according to proximal chromatin accessibility. **(B)** The statistical significance of a rank correlation between high, medium, and low magnitudes of chromatin discordance and high, medium, and low levels of differential gene expression, as assessed based on 10,000 permutations of differential expression and chromatin discordance. **(C)** Differential expression according to genes with broken TF motifs and relevant ChIP evidence compared with all genes as control.

The identified mutations were coupled to increases in the level of discordance in chromatin accessibility (Figure 1A) and therefore to increases in the likelihood of discordance at a given locus (Figure 1B). Mutations that decrease chromatin accessibility were more frequent than mutations that increase chromatin accessibility (Figure 1C). The impact of the mutations was stronger when they were located closer to the center of a region of open chromatin (Additional file 7) and when their density relative to the size of the open chromatin region (that is, number of mutations per base pair) was higher (Additional file 8). We then matched our mutation and chromatin discordance results with chromatin status modeling data from multilayer chromatin signatures such as open chromatin, histone modifications and TF binding [19]. Remarkably, poised promoters, more than any other regulatory states, exhibited the highest mutation rates (Figure 1D) and the highest discordance levels (Figure 1E), although they had the smallest site number and length. Importantly, a higher magnitude of mutational effects was observed when TF binding sites (TFBSs) located within open chromatin regions were disrupted (Figure 1B). Changes in TF binding have been shown to be a primary mechanism through which DNA polymorphisms can affect chromatin structure [11,13]. We found examples in which mutations in one twin sibling disrupted the binding motifs of key TFs implicated in immune cell development and function (ETS1, ELF1, PAX5, and RUNX) and decreased chromatin accessibility in an allele-specific manner (Figure 2).Our next concern was whether somatic mutation-derived chromatin discordance is associated with perturbations in gene expression. For that, we conducted expression microarray analyses and calculated the genome-wide expression divergence between twin siblings. We first compared the within-pair expression difference of genes showing promoter chromatin discordance with that of genes showing no chromatin discordance. Our results supported the significant effect of chromatin discordance on differential gene expression (Figure 3A). A rank correlation between high, medium, and low magnitudes of chromatin discordance and high, medium, and low levels of differential gene expression was statistically significant as assessed based on permutation tests (Figure 3B), thereby indicating that high accessibility tends to direct high expression levels. We then identified genes with somatic mutations that disrupted the TF motif and induced chromatin changes at proximal sites. TF-binding chromatin immunoprecipitation sequencing (ChIP-seq) data were incorporated simultaneously to confirm the binding of TFs to the corresponding motifs. We then found that genes with disrupted TF motifs exhibited a larger degree of differential expression than controls, when all genes were used as controls (Figure 3C). This gap in differential expression compared with the control was most striking when TF motifs were confirmed by the ChIP data (Figure 3C). These findings indicate that somatic mutations in proximal accessible chromatin regions can disrupt TF binding at the respective motifs and alter chromatin accessibility, which can influence the transcription of the connected genes.

**Figure 4:**
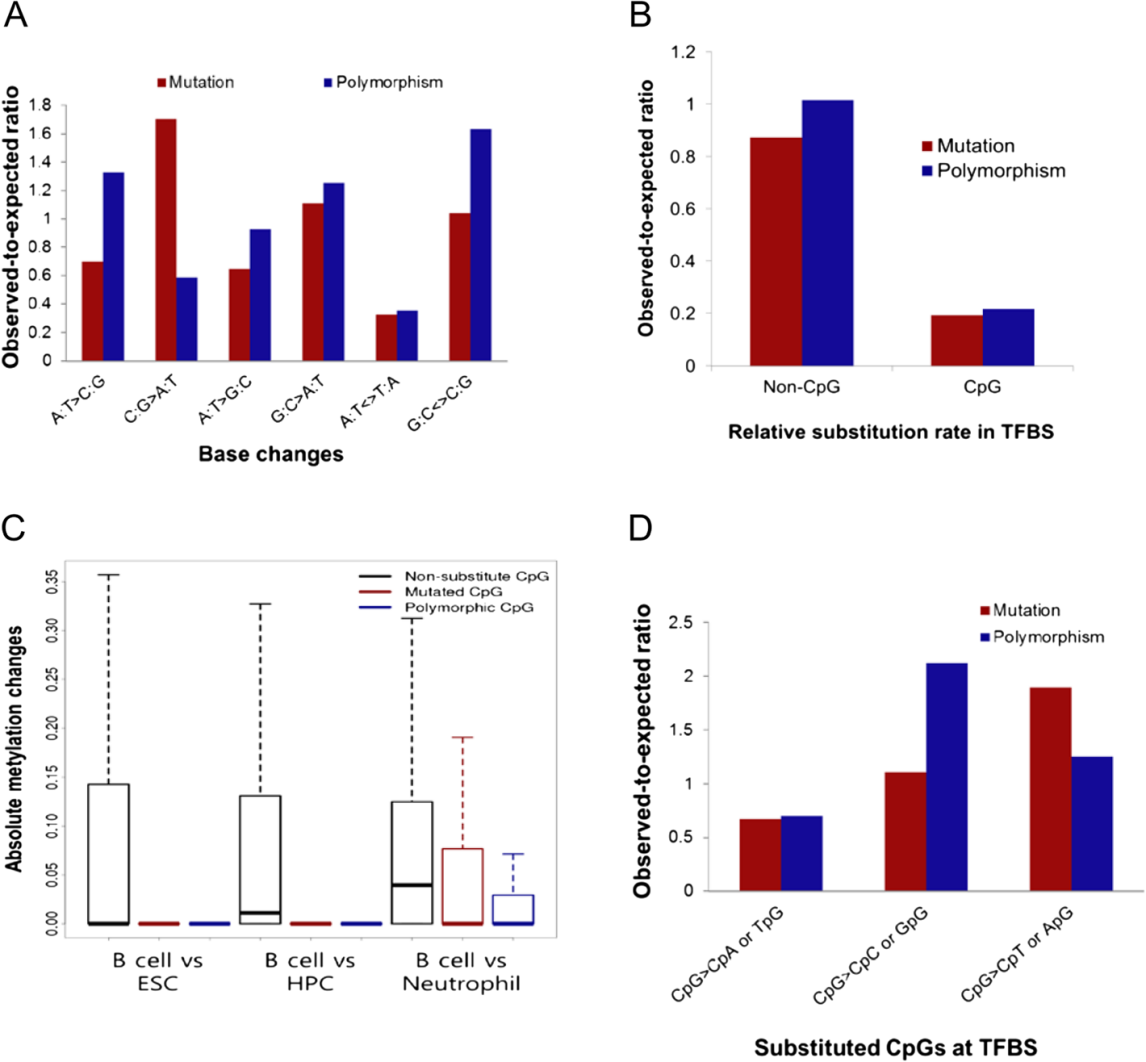
**Spectrum of base substitutions by mutation and polymorphism. (A)** Observed-to-expected ratios were defined as the ratio of the base change frequency of mutation (red) and polymorphism (blue) in TFBSs to the overall base change frequency in open chromatin. Only changes from the reference homozygote were considered. **(B)** Observed-to-expected ratios were defined as the ratio of the relative enrichment of substituted dinucleotides in TFBSs to the relative enrichment of all dinucleotides in TFBSs. **(C)** For each TFBS CpG, differential methylation levels between lymphocytes and other cell types (embryonic stem cells (ESC), hematopoietic progenitor cells (HPC), and neutrophils) were calculated. **(D)** Observed-to-expected ratios of base changes at TFBS CpGs were calculated as the ratio of the substitution frequency of mutation and polymorphism in TFBSs to the overall substitution frequency in open chromatin.

Somatic C:G > A:T transversions were observed as frequently as C:G > T:A transitions (red bars in Figure 4A). However, the occurrence of C:G > A:T polymorphisms was remarkably lower than that of the corresponding mutations (blue bars in Figure 4A), implying the influence of negative selection. The loss of C or G bases, particularly through changes that greatly alter their chemical structure, namely transversions, may exert large functional effects. Indeed, C:G > A:T transversions represented the base substitutions that were most commonly associated with chromatin discordance and inter-individual variation (Additional file 9). This likely explains why these mutations are subject to negative selection.

CpGs were substantially enriched in TFBSs relative to their overall frequency in the surrounding open chromatin (Additional file 10) or in the whole genome (Additional file 11). Nevertheless, CpGs were much less frequently mutated or polymorphic than expected (Figure 4B). The observed-to-expected ratio was approximately 0.2 for both mutation and polymorphism, which is approximately four-fold lower than the mean of the non-CpG group. We suspected that cytosine methylation might contribute to the lower than expected CpG substitution rates; therefore, we obtained the methylation levels for each in-TFBS CpG in human embryonic stem cells, hematopoietic progenitor cells, and mature cells from different lineages (neutrophils and lymphocytes) [20] and then estimated the changes in cytosine methylation that occur during B cell development. When comparing lymphocytes to other cell types, non-substituted CpGs showed a larger magnitude of differential methylation; however, mutated and polymorphic CpGs displayed no significant methylation changes (Figure 4C). Similar patterns were obtained when the average TFBS methylation levels were used for comparison (Additional file 12). The role of DNA methylation in controlling differentiation-associated TF binding has been demonstrated previously [21,22]. Therefore, we suggest that TFBS CpGs whose methylation is specifically regulated during cellular differentiation maintain low mutation rates due to negative selection acting within organisms. There were no distinct signs of predominant 5-methylcytosine mutations (that is, mCpG > TpG; Figure 4D), suggesting that the functional importance of regulatory CpGs can counteract the high mutation rate of 5-methylcytosines. The differences in methylation levels between the differentiated cell types (B cells versus neutrophils) showed weaker effects (Figure 4C; Additional file 12), highlighting the importance of methylation regulation during early stages of development. Taken together, these findings suggest that the landscape of CpG variations may be shaped by developmental, rather than evolutionary, processes.

In this work, sequencing reads were derived only from open chromatin regions, thereby enabling in-depth sequencing. Moreover, the functional validity of the putative mutations was supported by the relevant chromatin and transcription changes. A completely independent dataset based on a different experimental approach (DNase I hypersensitivity), SNP calling (HapMap genotyping and 1000 Genomes sequencing), and population (Yoruba) [13] confirmed selective constraints against transversions (Additional file 13), against changes in GpC dinucleotides (Additional file 14), and against substitutions at differentiation-associated CpG methylation sites (Additional file 15). Furthermore, the overall consistency between the spectra of the mutations and polymorphisms suggests that the majority of the mutations identified in the examined cell lines reflect *in vivo* characteristics.

The concept of ‘variability genes’ was suggested based on the finding that the Kidd blood group locus is associated with within-pair differences in the total cholesterol level when serum lipid levels were examined across twin pairs with different genotypes [23]. Therefore, variability genes refer to genotypes that are associated with the variance of a trait rather than with the level of a trait, thereby implying that within-pair variability in MZ twins can be used to study genetic-epigenetic interactions [24]. In this regard, we attempted to identify genetic polymorphisms that are shared by twin siblings and are associated with within-pair differences in chromatin accessibility. In other words, we sought to find the cases in which certain chromatin sites are more differentially accessible between twin siblings who share a particular allele than between other siblings with different alleles.

**Figure 5:**
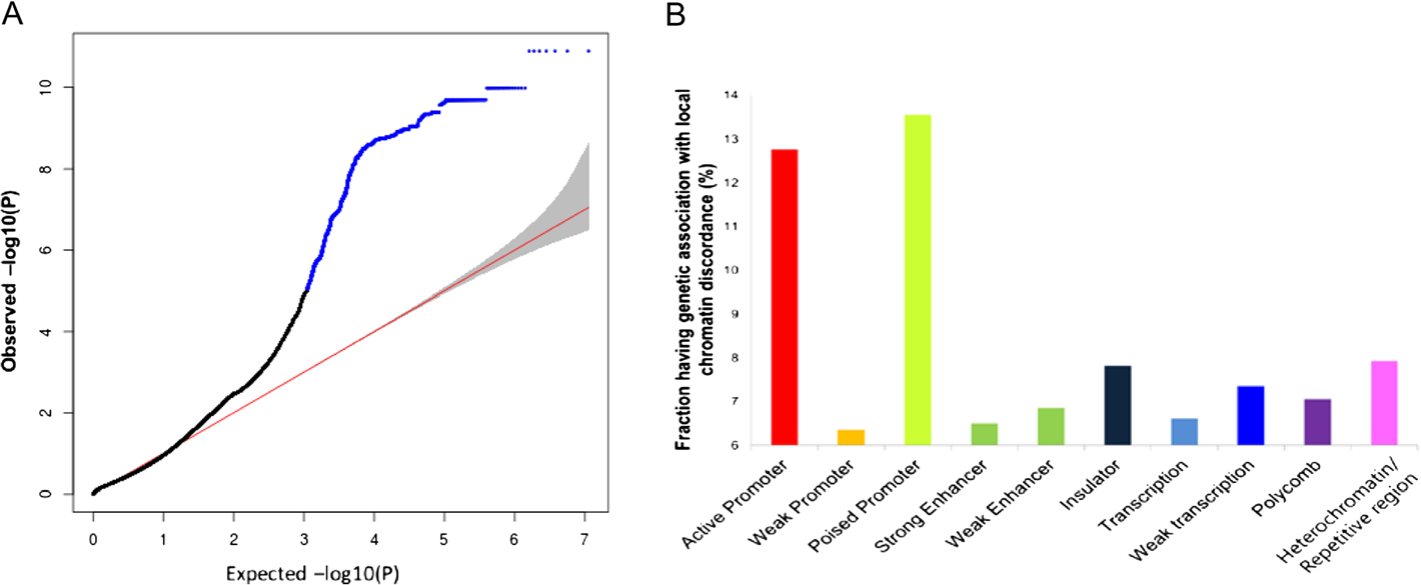
**Interplay between genetic and non-genetic factors causing chromatin discordance. (A)** Quantile-quantile plot of *P* values for local (<1 Mb) associations from the QTL mapping of chromatin discordance. The 5^th^ and 95^th^ percentiles of the beta distribution of the *P* values is shown (gray shading). Significant associations (FDR = 0.01) are denoted in blue. **(B)** The percentage of chromatin-state domains harboring the chromatin regions that are discordant depending on a local genotype. The number of such domains was divided by the total number of the domains containing the open chromatin regions used in our QTL mapping.

To this end, we performed QTL mapping by associating the within-pair differences in chromatin accessibility with the genotypes shared by each twin pair as determined using the Affymetrix SNP arrays. As previously suggested [23], normalized differences in chromatin accessibility were used instead of absolute differences (see Materials and methods) to rule out the possibility that the trait level itself is reflected in the degree of difference. Because within-pair chromatin differences are very low in most cases, the chromatin loci with the highest between-pair variances in chromatin discordance (for example, the top 1%) were selected and used for QTL mapping. At a false discovery rate (FDR) of 0.01, a total of 10,195 local (*cis*) associations were identified for 1,325 chromatin loci (Figure 5A). In particular, poised promoters and active promoters showed the highest levels of enrichment for such associations (Figure 5B), which was similar with the case of somatic mutations. Within-pair differences in chromatin states can be caused by epigenetic factors, such as histone modifications or the expression levels of chromatin regulators or TFs, that reflect different histories of environmental exposure between twin siblings. These epigenetic differences will be exposed by differential TF binding in one sibling in the form of differential chromatin accessibility but be masked by genetically low TF binding to the TFBS in the other sibling.

Notably, poised promoters presented high levels of chromatin discordance between twin siblings (Figure 1E) either in association with a high frequency of somatic mutations (Figure 1D) or via the mechanisms of the variability genotypes (Figure 5B). Poised transcriptional elements are critical in defining cellular responses to environmental or developmental cues. In the context of cellular differentiation, bivalent histone modifications (for example, H3K4me3 and H3K27me3) may silence developmental genes in embryonic stem cells while keeping these genes poised for activation during later stages of development [25-27]. Our findings suggest that promoters that are currently inactive but are poised to be activated following particular stimuli exhibit high levels of chromatin discordance, due to either somatic mutations or genetic-epigenetic interactions, and are responsible for differential regulatory responses to environmental or developmental cues in twin siblings.

By leveraging MZ twin samples, we were able to identify somatic mutations as within-pair genetic differences and provide new insight into their effect on chromatin accessibility for the first time. Previous studies concentrated on genetic polymorphisms residing in accessible chromatin [9-14]. Three recent papers [28-30] went further to confirm a causal role of polymorphisms in modulating epigenetic and transcription mechanisms through the regulation of TF binding. We expected that, from a mechanistic viewpoint, somatic mutations would affect transcription in a similar manner to polymorphisms. Indeed, we discovered that somatic mutations could lead to differential gene expression through chromatin discordance and TF motif disruption. The differences between mutations and polymorphisms also provided new insight. For example, the critical substitution, namely C:G > A:T transversion, was less enriched for polymorphisms than for somatic mutations, implying the action of negative selection.

Previous twin studies primarily focused on differences in DNA methylation [15-17]. Our study is the first to profile genetic differences between multiple MZ twins and examine their linkage with chromatin discordance and differential transcription. We expect that follow-up twin studies will also address genetic discordance in association with the epigenetic differences in question. Heterogeneity in the cell population may have affected our detection of genetic and epigenetic discordance between MZ twin siblings. However, mutation presence and chromatin discordance were associated based on the same FAIRE-seq data of the same cell population. The genetic and associated epigenetic differences should have arisen at the identical lineage depth of cell differentiation, thereby mitigating the heterogeneity problem in the context of genetic-epigenetic association. We also found cases in which somatic mutations in TFBSs altered chromatin accessibility between twin pairs with discordant allergic traits. Several of the TFs in these cases were reported to have immunological functions; therefore, we speculated that disruptions in the binding of immune-related TFs could bring about differences of chromatin accessibility and allergic phenotypes. This raises the possibility that chromatin discordancy related to somatic mutations could affect phenotypic diversity, but identifying a direct connection between chromatin accessibility and the target trait requires further study.

## Conclusions

In this work, we performed targeted deep sequencing for open chromatin and gene expression microarray experiments across the genomes of a fairly large number of MZ twins. Our integrative analyses identified somatic mutations causing chromatin discordance mainly via the disruption of TFBSs. The spectrum of somatic and genetic sequence variations underlying discordant open chromatin suggested selection pressures against C:G > A:T transversions. Of note, CpGs located in TFBSs were found to be mutated or polymorphic only one-fifth as often as expected. In particular, those CpGs whose methylation is specifically regulated during cellular differentiation appeared to be protected from the high mutation rates associated with 5′-methylcytosines, thereby implying that the spectrum of CpG variations may be shaped fully at the developmental level but not through natural selection. Our association mapping of within-pair chromatin differences identified cases in which only twin siblings with a particular genotype exhibited chromatin discordance at the relevant locus, demonstrating that epigenetic differences can bring about regulatory variations through interactions with genetic factors. Remarkably, poised promoters presented high levels of chromatin discordance in association with either somatic mutations or genetic-epigenetic interactions, reflecting their role in inducing differential regulatory responses to environmental or developmental cues in twin siblings. In conclusion, our observations illustrate how somatic mutations and genetic polymorphisms may contribute to regulatory, and ultimately phenotypic, discordance.

## Materials and methods

### MZ twin samples

This study was approved by the Institutional Review Board of the Korea National Institute of Health (KNIH) and Korea Advanced Institute of Science and Technology (KAIST). Written informed consent was obtained from all individuals. Peripheral blood mononuclear cells were extracted from 36 pairs of MZ twins, aged 30 to 60 years, who were discordant for immunological traits mostly involving allergic symptoms (Additional file 2). Lymphoblastoid cells were generated as previously described [31].

### Open chromatin sequencing

Sequencing of accessible chromatin regions was performed as described previously [2,7-9]. Following quality control of the DNA library, 50-bp single-end Illumina HiSeq2000 sequencing was performed. CASAVA-1.8.2 was used to align sequence tags to the reference human genome (hg19) using the default option, which disallowed mismatches. The mean Phred quality (Q) score of the bases in the passed-filter reads was averaged at 36.2 across the 72 samples (Additional file 1). The average percentage of the bases with Q ≥ 30 in the passed-filter reads was 91.9% (Additional file 1).

### Identification of open chromatin regions

To identify genomic regions enriched for open chromatin tags, we employed the Zero Inflated Negative Binomial Algorithm (ZINBA) [32]. Tags were extended 134 bp as recommended by the ZINBA program for the prediction of open chromatin peaks. An FDR threshold of 0.05 was used. For covariates, the G/C content, the proportion of mappable bases (mappability score), and the local background estimate were taken into account. Open chromatin regions were reported to encompass 1 to 2% of the genome [32]. In line with this, the peaks we identified covered 2.01% of the hg19 genome on average. The number of tags mapped to these regions is summarized in Additional file 1. The effective sequencing depth was computed by dividing the total number of bases read by the tags mapped to the identified peaks (the number of reads multiplied by the read length) by the total base pairs covered by the peaks (that is, 2.01% on average). For confirmation, we obtained genomic regions enriched for DNase I hypersensitivity as identified by F-Seq [33] and those enriched for FAIRE signals as called using ZINBA [32] from the ENCODE Open Chromatin Synthesis track of the UCSC Genome Browser [34] for the GM12878 lymphoblastoid cell line.

### Quantification and normalization of chromatin accessibility

For the quantification of chromatin accessibility, the open chromatin peaks identified from each of the 72 samples were merged into meta-peaks using BEDTools [35]. For each sample, the accessibility signals were normalized as previously suggested [36,37]; the number of sequenced reads mapped to each meta-peak was counted and divided by the length of the meta-peak, which was then calibrated by the ratio of the total read count to the genome size. This metric measures the enrichment of tags within a given open chromatin locus relative to the whole genome. We also employed a quantile-quantile normalization method to remove the effect of between-sample variations by using the Quantile Normalization package of R. Chromatin discordance was calculated for each locus as the quantitative difference between the normalized chromatin accessibility measures of twin siblings. The absolute difference, |*X*_1_ - *X*_2_|, was also obtained, wherein *X*_1_ and *X*_2_ indicate chromatin accessibility in each sibling of a twin pair. While this provides a direct measure of chromatin differences, it can cause a bias toward highly accessible chromatin sites such as active promoters or strong enhancers. Therefore, we normalized this measure as |*X*_1_ - *X*_2_|/(*X*_1_ + *X*_2_) and used the normalized difference particularly when comparing different regulatory states. Discordant chromatin sites were determined based on effect size (that is, when the within-pair fold change was greater than four) or based on variation analysis (that is, when the within-pair variance in chromatin accessibility was greater than the between-pair variance).

### Identification of mutations and polymorphisms

Somatic mutations were defined as within-pair single nucleotide differences. We employed the method suggested by VarScan [38,39] for the statistical test of the number of aligned reads supporting each allele. The allele frequency obtained at every mapped position was subjected to Fisher’s exact test. To determine the direction of base changes, we considered the cases in which one sibling in a given pair is a reference homozygote. Only high-confidence calls by VarScan were considered. We further raised the threshold by selecting those supported by 10 or more high-quality aligned reads on both strands and with a *P* value from Fisher’s exact test lower than 1 × 10^-3^. For the identification of genetic polymorphisms (SNPs), we employed the germline variant-calling functionality of VarScan to analyze variants that were not significantly different between twin siblings (that is, ones that did not pass the mutation identifier). Variant calls were filtered when >90% of the supporting reads in each sample came from only one strand in order to remove alignment-related artifacts. We considered autosomal genomic positions that were covered by at least one read in all of the 72 samples. To be identified as a polymorphism, a minimum allele frequency of 1% among all the reads across the samples was required. Association mapping was conducted between the identified SNPs and the normalized accessibility signals across the 72 samples based on simple linear regression by using the scipy.stats module of SciPy. Significant associations were chosen at the FDR of 0.05 with *P* value adjustment by using the fdrtool package of R.

### Array genotyping and validation

Genomic DNA (500 ng) was isolated from the peripheral blood cells of one sibling from each twin pair and genotyped on the Affymetrix Genome-Wide Human SNP array 6.0. Low-concentration genomic DNA was amplified prior to genotyping according to the manufacturer’s protocol (QIAGEN). The Bayesian Robust Linear Modeling using Mahalanobis Distance (RLMM) algorithm was used to perform genotype calling of 516,188 SNPs [40]. SNP imputation was carried out using IMPUTE (version 2) as described previously [19,41] on the basis of the genotypes of 286 Asian individuals from the 1000 Genomes Project (CHB [ASN] (n = 97) - Han Chinese in Beijing, China; CHS [ASN] (n = 100) - Han Chinese South; JPT [ASN] (n = 89) - Japanese in Tokyo, Japan) as a reference panel. Validation was performed for autosomal chromosomes to check whether our genotyping based on open chromatin sequencing would reproduce the microarray genotypes. To assess the effect of technical issues such as possible biases in the allele frequencies of open chromatin sequences, we interrogated the available array data to determine whether the genotype of each individual is a reference homozygote or variant homo-/hetero-zygote for each locus of the identified mutations or polymorphisms from the open chromatin sequencing. For polymorphic loci, the genotypes of all available individuals were examined. For the mutation loci, the available array genotype of one of the relevant siblings was compared with the sequencing-based genotype when the mutant allele matched one of the common alleles on the array. To increase the overlap between the platforms, we used all of the high-confidence mutations without using a *P*-value threshold. Sixty-six percent of the individual genotypes at individual loci were reproducible (Additional file 5). Among the unmatched cases (Additional file 6) were those in which the array genotype indicated a homozygous variant (for example, A/A) while the open chromatin sequences seemed to support a heterozygous variant genotype (for example, A/G). Because the sequencing data support the existence of the reference allele (that is, G), it is most likely that the reference allele was not detected via array hybridization. Including those cases (denoted in blue in Additional file 6) in the matched list increased the concordance rate to 68.6%. There may also be cases in which the subject is actually a heterozygous variant (for example, A/G) when the captured open-chromatin sequences might be biased toward the allele that increases chromatin accessibility (that is, A), leading to an apparently homozygous variant genotype (that is, A/A). These instances are shown in red in Additional file 6. Because we treated variant homozygotes and heterozygotes equally as one instance of variation, this type of mismatch did not affect the spectrum of nucleotide variations that we examined in this work. Permitting these cases increased the concordance rate to 73.6%. Taken together, the overall concordance of variant detection was 76.2%. It should be noted that, for validation purposes, we did not use *P*-value filtering. For our main analysis, we further filtered mutations using a *P*-value threshold of 1 × 10^-3^.

### Dinucleotide counting and typing

Complementary base pairs were concatenated by ‘:’ (for example, A:T or C:G) and dinucleotides were connected by ‘p’ (for example, CpG, GpC, and so on). For the typing of mutations and polymorphisms arising at different dinucleotides, we extracted three consecutive bases centered on each of the identified variants (mutations or polymorphisms) using BEDTools [35] and counted the dinucleotide composed of the -1 base and the variant (-1pV) alongside the dinucleotide composed of the variant followed by the +1 base (Vp + 1). The number of dinucleotides themselves was counted in the same manner for all different trinucleotides. Changes on one strand were treated equally with complementary changes on the opposite strand. For example, the change from the trinucleotide GpApA (reference) to GpCpA (variant) was treated as 5′-GpA-3′ to 5′-GpC-3′ on one strand or 5′-TpC-3′ to 5′-GpC-3′ on the other strand, and simultaneously as 5′-ApA-3′ to 5′-CpA-3′ on one strand or 5′-TpT-3′ to 5′-TpG-3 on the other stand. The number of observed substitutions was counted for each type of dinucleotide by combining all possible changes from the dinucleotide type (for example, for ApA, the instances of ApA > CpA, ApA > GpA, ApA > TpA, ApA > ApC, ApA > ApG, ApA > ApT were combined). By following the above procedure, the number of palindromic dinucleotides (that is, CpG, GpC, ApT, and TpA), as well as the occurrence of mutations and polymorphisms in such dinucleotides, was double-counted to provide fair comparison with the other dinucleotides.

### Expression microarray analysis

Total RNA was extracted from lymphoblastoid cells using RNeasy Mini Kit (QIAGEN, Chatsworth, CA, USA) according to the manufacturer’s instructions. The yield and purity of the extracted RNA were evaluated by A260/A280 measurement and agarose gel electrophoresis. Gene expression levels were measured for the 72 MZ twin samples on the Illumina Human HT-12 chip. The raw intensities were log2 transformed and normalized by using the VST-quantile normalization method of the lumi R packages (v.14.01).

### Transcription factor binding site data

A total of approximately 4 million evolutionarily conserved binding sites for 250 TFs, as inferred from the Transfac Matrix Database (v7.0) [42,43], were obtained from the Human/Mouse/Rat Conserved Transcription Factor Binding Sites track of the UCSC Genome Browser. The sites of *in vivo* TF binding were obtained from the ENCODE Transcription Factor Binding tracks. All data available for the GM12878 cells were generated either by the Hudson Alpha Institute for Biotechnology (HAIB) or by Stanford/Yale/USC/Harvard (SYDH). We first discovered regions enriched for TF binding by using the peak finding functionality of the Homer package [44]. We then located the peak summit as the position at which the maximum number of ChIP-seq tags overlapped within the given region. ChIP-seq peaks in which less than 80% of the sequencing tags covered the peak summit were discarded. In this manner, we were able to select peaks that were most likely to contain the focused binding site of a single TF. However, this set of TFBSs was not used for mutation typing because the actual binding motifs of TFs are hard to predict based on the library fragments, which are only 200 bp on average. Additionally, we searched the open chromatin peaks for enriched TF motifs by using the find MotifsGenome function of Homer [44]. Homer’s library of known motifs was screened against the target and background sequences for enrichment, and motifs enriched with a *P* < 0.05 based on the binomial distribution were selected. For *de novo* motif finding, motifs of 8, 10, and 12 bp in length were screened for enrichment.

### Dinucleotide enrichment in transcription factor binding sites

To measure the relative enrichment of each dinucleotide in TFBSs, compared with background sequences, we counted each dinucleotide located in a TFBS and its surrounding open chromatin region as described above. The total occurrence of the dinucleotide in a TFBS was divided by its occurrence in the surrounding open chromatin regions. To control for differences in the sizes of TFBSs and open chromatin regions, the number of all the dinucleotides in the TFBSs was divided by the number of all the dinucleotides in the surrounding open chromatin regions. The ratio obtained for each specific dinucleotide was then divided by the ratio obtained for all possible dinucleotides. This ratio of ratios then served as the measure of the relative enrichment of dinucleotides in TFBSs (Additional file 10). We also repeated this test using the whole genome as the background, instead of just the open chromatin regions, to adjust for the differential frequencies of different dinucleotides across the whole genome (Additional file 11). We then repeated the same procedure for mutated dinucleotides and polymorphic dinucleotides. Specifically, the relative enrichment of substituted dinucleotides in TFBSs was obtained as (Number of given substituted dinucleotides in TFBSs/Number of given substituted dinucleotides in surrounding open chromatin)/(Number of all substituted dinucleotides in TFBSs/Number of all substituted dinucleotides in open chromatin). This ratio was divided by the relative enrichment of the dinucleotides themselves as described above, namely as (Number of given dinucleotides in TFBSs/Number of given dinucleotides in surrounding open chromatin)/(Number of all dinucleotides in TFBSs/Number of all dinucleotides in open chromatin). This metric was used to estimate the observed-to-expected ratio of mutations or polymorphisms.

### Quantitative trait loci mapping

The normalized chromatin difference, |*X*_1_ - *X*_2_|/(*X*_1_ + *X*_2_), was obtained using X1 and X2 to indicate the chromatin accessibility in each sibling of a twin pair. Because the within-pair chromatin differences are very low in most cases, the chromatin loci with the highest between-pair variances in chromatin discordance (that is, the top 1%) were selected. We used the genotypes obtained from the Affymetrix Genome-Wide Human SNP array 6.0, which were imputed as described above, and filtered at the minor allele frequency of 1%. A simple linear regression model from the Matrix eQTL package was applied [45]. *Cis*-associations were defined as a distance of less than 1 Mb between the genetic marker and the associated chromatin site.

### Other data

Cytosine methylation levels were derived from whole-genome shotgun bisulfite sequencing data from human embryonic stem cells, hematopoietic stem/progenitor cells, B lymphocytes, and neutrophils [20]. Data for different regulatory states in the GM12878 lymphoblastoid cell line were downloaded from the Chromatin State Segmentation by HMM from the ENCODE/Broad track of the UCSC Genome Browser. DNase I hypersensitive sites in 70 Yoruba lymphoblastoid cell lines [13] were obtained from [46]. The genotypes given by the 1000 Genomes YRI (Yoruba in Ibadan) high coverage cohort, YRI HapMap panel, and 1000 Genomes YRI low coverage cohort were used in the order of preference depending on data availability [13]. The combined final genotype for each locus in each individual was obtained from [47]. We selected SNPs falling within DNase I hypersensitive sites in each sample and performed polymorphism typing as described above.

### Data access

The expression profile and FAIRE-seq data from this study have been submitted to the NCBI Gene Expression Omnibus under accession numbers [GEO:GSE53822 and GSE44742].

## Abbreviations

bp: base pair; ChIP: chromatin immunoprecipitation; FAIRE: formaldehyde-assisted isolation of regulatory elements; FDR: false discovery rate; MZ: monozygotic; QTL: quantitative trait locus; SNP: single-nucleotide polymorphism; TF: transcription factor; TFBS: transcription factor binding site.

## Competing interests

The authors declare that they have no competing interests.

## Authors’ contributions

KK and HJB performed the data analyses and drafted the manuscript. JS, KL, and MY carried out the FAIRE-seq, genotype array, and expression array experiments. KK participated in the FAIRE-seq experiments. HJB participated in the genotype array and expression array experiments. SCK assisted the data analyses. KP, SBC, and JKC conceived the study. SBC and JKC supervised the study and wrote the manuscript. All authors read and approved the final manuscript.

## Supplementary Material

Additional file 1 Information of the MZ twin FAIRE-seq data. Click here for file

Additional file 2 Clinical information of the MZ twins used in this work. Click here for file

Additional file 3 Density plot for genome-wide correlation coefficients of chromatin accessibility between twin siblings and between unrelated individuals. Click here for file

Additional file 4 **The number of open chromatin regions we identified that overlap with peaks identified from the public FAIRE-seq and DNase-seq data for the GM12878 lymphoblastoid cells.** Our FAIRE regions were extended 10 to 200 bp before overlapping. Approximately 83% of the FAIRE regions that we found to overlap with the public FAIRE data were confirmed by the public DNase I data. Click here for file

Additional file 5 List of sequencing variants whose genotype is confirmed by Affymetrix SNP array 6.0. Click here for file

Additional file 6 **List of sequencing variants whose genotype is in conflict with the Affymetrix SNP array 6.0 genotype.** Red/blue coloring indicates cases in which a sequencing variant calling matches an array variant calling in terms of genotype identity irrespective of homo-/hetero-zygosity. Click here for file

Additional file 7 Within-pair differences in chromatin accessibility as a function of the distance between the mutation and the center of the chromatin region. Click here for file

Additional file 8 Within-pair differences in chromatin accessibility according to the number of mutations per base pair as an estimate of the density of mutations relative to the size of the open chromatin region. Click here for file

Additional file 9 Observed-to-expected ratios of the substitution frequency of TFBS mutations and polymorphisms that were associated with chromatin discordance and inter-individual variation, respectively. Click here for file

Additional file 10 **The relative enrichment of dinucleotides in TFBSs.** The ratio of the number of the specified dinucleotides in TFBSs to the number in the surrounding chromatin regions was divided by the ratio for all the different dinucleotides. Click here for file

Additional file 11 **The relative enrichment of dinucleotides in TFBSs.** The ratio of the number of each dinucleotide in TFBSs to the number in the whole genome was divided by the ratio of the number of all the different dinucleotides in TFBSs to the number in the whole genome. Click here for file

Additional file 12 **Differential methylation levels between B lymphocytes and other cell types (embryonic stem cells (ESC), hematopoietic progenitor cells (HPC), and neutrophils) for each TFBS CpG.** The average of cytosine methylations in TFBSs was obtained and plotted. Click here for file

Additional file 13 **The frequency of different types of polymorphisms arising in TFBSs as identified in this work (dark blue) and in the previous work by Degner *et al*. **[13]** (sky blue).** Only changes from the reference homozygote were considered. Click here for file

Additional file 14 **The frequency of TFBS dinucleotides containing polymorphisms identified in this work (dark blue) and in the previous work by Degner *et al*. **[13]** (sky blue).** Click here for file

Additional file 15 **Left: for the TFBS polymorphisms identified by Degner *et al*. **[13]**, observed-to-expected ratios were obtained as the ratio of the relative enrichment of polymorphic dinucleotides in TFBSs to the relative enrichment of all dinucleotides in TFBSs.** Right: for each TFBS CpG, differential methylation levels between B lymphocytes and other cell types (embryonic stem cells (ESC), hematopoietic progenitor cells (HPC), and neutrophils) were calculated. Click here for file

## References

[B1] Boyle AP Davis S Shulha HP Meltzer P Margulies EH Weng Z Furey TS Crawford GE High-resolution mapping and characterization of open chromatin across the genome Cell 2008 132 311 322 10.1016/j.cell.2007.12.014 18243105 PMC2669738

[B2] Song L Zhang Z Grasfeder LL Boyle AP Giresi PG Lee B-K Sheffield NC Graf S Huss M Keefe D Liu Z London D McDaniell RM Shibata Y Showers KA Simon JM Vales T Wang T Winter D Zhang Z Clarke ND Birney E Iyer VR Crawford GE Lieb JD Furey TS Open chromatin defined by DNaseI and FAIRE identifies regulatory elements that shape cell-type identity Genome Res 2011 21 1757 1767 10.1101/gr.121541.111 21750106 PMC3202292

[B3] Berchowitz LE Hanlon SE Lieb JD Copenhaver GP A positive but complex association between meiotic double-strand break hotspots and open chromatin in Saccharomyces cerevisiae Genome Res 2009 19 2245 2257 10.1101/gr.096297.109 19801530 PMC2792181

[B4] Audit B Zaghloul L Vaillant C Chevereau G d’Aubenton-Carafa Y Thermes C Arneodo A Open chromatin encoded in DNA sequence is the signature of ‘master’ replication origins in human cells Nucleic Acids Res 2009 37 6064 6075 10.1093/nar/gkp631 19671527 PMC2764438

[B5] Thurman RE Rynes E Humbert R Vierstra J Maurano MT Haugen E Sheffield NC Stergachis AB Wang H Vernot B Garg K John S Sandstrom R Bates D Boatman L Canfield TK Diegel M Dunn D Ebersol AK Frum T Giste E Johnson AK Johnson EM Kutyavin T Lajoie B Lee BK Lee K London D Lotakis D Neph S The accessible chromatin landscape of the human genome Nature 2012 489 75 82 10.1038/nature11232 22955617 PMC3721348

[B6] Neph S Vierstra J Stergachis AB Reynolds AP Haugen E Vernot B Thurman RE John S Sandstrom R Johnson AK Maurano MT Humbert R Rynes E Wang H Vong S Lee K Bates D Diegel M Roach V Dunn D Neri J Schafer A Hansen RS Kutyavin T Giste E Weaver M Canfield T Sabo P Zhang M Balasundaram G An expansive human regulatory lexicon encoded in transcription factor footprints Nature 2012 489 83 90 10.1038/nature11212 22955618 PMC3736582

[B7] Giresi PG Kim J McDaniell RM Iyer VR Lieb JD FAIRE (Formaldehyde-Assisted Isolation of Regulatory Elements) isolates active regulatory elements from human chromatin Genome Res 2007 17 877 885 10.1101/gr.5533506 17179217 PMC1891346

[B8] Waki H Nakamura M Yamauchi T Wakabayashi K-i Yu J Hirose-Yotsuya L Take K Sun W Iwabu M Okada-Iwabu M Fujita T Aoyama T Tsutsumi S Ueki K Kodama T Sakai J Aburatani H Kadowaki T Global mapping of cell type–specific open chromatin by FAIRE-seq reveals the regulatory role of the NFI family in adipocyte differentiation PLoS Genet 2011 7 e1002311 10.1371/journal.pgen.1002311 22028663 PMC3197683

[B9] Gaulton KJ Nammo T Pasquali L Simon JM Giresi PG Fogarty MP Panhuis TM Mieczkowski P Secchi A Bosco D Berney T Montanya E Mohlke KL Lieb JD Ferrer J A map of open chromatin in human pancreatic islets Nat Genet 2010 42 255 259 10.1038/ng.530 20118932 PMC2828505

[B10] Smith AJ Howard P Shah S Eriksson P Stender S Giambartolomei C Folkersen L Tybjærg-Hansen A Kumari M Palmen J Hingorani AD Talmud PJ Humphries SE Use of allele-specific FAIRE to determine functional regulatory polymorphism using large-scale genotyping arrays PLoS Genet 2012 8 e1002908 10.1371/journal.pgen.1002908 22916038 PMC3420950

[B11] Lee K Kim SC Jung I Kim K Seo J Lee H-S Bogu GK Kim D Lee S Lee B Choi JK Genetic landscape of open chromatin in yeast PLoS Genet 2013 9 e1003229 10.1371/journal.pgen.1003229 23408895 PMC3567132

[B12] McDaniell R Lee B-K Song L Liu Z Boyle AP Erdos MR Scott LJ Morken MA Kucera KS Battenhouse A Keefe D Collins FS Willard HF Lieb JD Furey TS Crawford GE Iyer VR Birney E Heritable individual-specific and allele-specific chromatin signatures in humans Science 2010 328 235 239 10.1126/science.1184655 20299549 PMC2929018

[B13] Degner JF Pai AA Pique-Regi R Veyrieras JB Gaffney DJ Pickrell JK De Leon S Michelini K Lewellen N Crawford GE Stephens M Gilad Y Pritchard JK DNase I sensitivity QTLs are a major determinant of human expression variation Nature 2012 482 390 394 10.1038/nature10808 22307276 PMC3501342

[B14] Maurano MT Humbert R Rynes E Thurman RE Haugen E Wang H Reynolds AP Sandstrom R Qu H Brody J Shafer A Neri F Lee K Kutyavin T Stehling-Sun S Johnson AK Canfield TK Giste E Diegel M Bates D Hansen RS Neph S Sabo PJ Heimfeld S Raubitschek A Ziegler S Cotsapas C Sotoodehnia N Glass I Sunyaev SR Systematic localization of common disease-associated variation in regulatory DNA Science 2012 337 1190 1195 10.1126/science.1222794 22955828 PMC3771521

[B15] Bell JT Spector TD A twin approach to unraveling epigenetics Trends Genet 2011 27 116 125 10.1016/j.tig.2010.12.005 21257220 PMC3063335

[B16] Fraga MF Ballestar E Paz MF Ropero S Setien F Ballestar ML Heine-Suñer D Cigudosa JC Urioste M Benitez J Boix-Chornet M Sanchez-Aguilera A Ling C Carlsson E Poulsen P Vaag A Stephan Z Spector TD Wu YZ Plass C Esteller M Epigenetic differences arise during the lifetime of monozygotic twins Proc Natl Acad Sci U S A 2005 102 10604 10609 10.1073/pnas.0500398102 16009939 PMC1174919

[B17] Kaminsky ZA Tang T Wang S-C Ptak C Oh GHT Wong AHC Feldcamp LA Virtanen C Halfvarson J Tysk C McRae AF Visscher PM Montgomery GW Gottesman II Martin NG Petronis A DNA methylation profiles in monozygotic and dizygotic twins Nat Genet 2009 41 240 245 10.1038/ng.286 19151718

[B18] Liu Y Aryee MJ Padyukov L Fallin MD Hesselberg E Runarsson A Reinius L Acevedo N Taub M Ronninger M Shchetynsky K Scheynius A Kere J Alfredsson L Klareskog L Ekström TJ Feinberg AP Epigenome-wide association data implicate DNA methylation as an intermediary of genetic risk in rheumatoid arthritis Nat Biotechnol 2013 31 142 147 10.1038/nbt.2487 23334450 PMC3598632

[B19] Ernst J Kheradpour P Mikkelsen TS Shoresh N Ward LD Epstein CB Zhang X Wang L Issner R Coyne M Ku M Durham T Kellis M Bernstein BE Mapping and analysis of chromatin state dynamics in nine human cell types Nature 2011 473 43 49 10.1038/nature09906 21441907 PMC3088773

[B20] Hodges E Molaro A Santos COD Thekkat P Song Q Uren PJ Park J Butler J Rafii S McCombie WR Smith AD Hannon GJ Directional DNA methylation changes and complex intermediate states accompany lineage specificity in the adult hematopoietic compartment Mol Cell 2011 44 17 28 10.1016/j.molcel.2011.08.026 21924933 PMC3412369

[B21] Bock C Beerman I Lien WH Smith ZD Gu H Boyle P Gnirke A Fuchs E Rossi DJ Meissner A DNA methylation dynamics during in vivo differentiation of blood and skin stem cells Mol Cell 2012 47 633 647 10.1016/j.molcel.2012.06.019 22841485 PMC3428428

[B22] Stadler MB Murr R Burger L Ivanek R Lienert F Schöler A Nimwegen E Wirbelauer C Oakeley EJ Gaidatzis D Tiwari VK Schübeler D DNA-binding factors shape the mouse methylome at distal regulatory regions Nature 2011 480 490 495 22170606 10.1038/nature10716

[B23] Hoffman H Torres WE Ernst RD Paleoradiology: advanced CT in the evaluation of nine Egyptian mummies Radiographics 2002 22 377 385 10.1148/radiographics.22.2.g02mr13377 11896227

[B24] Gross AW Aprille JR Ernst SG Identification of human mitochondrial DNA fragments corresponding to the genes for ATPase, cytochrome C oxidase, and nine tRNAs in a denaturing gradient gel electrophoresis system Anal Biochem 1994 222 507 510 10.1006/abio.1994.1526 7864382

[B25] Barski A Cuddapah S Cui K Roh T-Y Schones DE Wang Z Wei G Chepelev I Zhao K High-resolution profiling of histone methylations in the human genome Cell 2007 129 823 837 10.1016/j.cell.2007.05.009 17512414

[B26] Wang Z Zang C Rosenfeld JA Schones DE Barski A Cuddapah S Cui K Roh TY Peng W Zhang MQ Zhao K Combinatorial patterns of histone acetylations and methylations in the human genome Nat Genet 2008 40 897 903 10.1038/ng.154 18552846 PMC2769248

[B27] Bernstein BE Mikkelsen TS Xie X Kamal M Huebert DJ Cuff J Fry B Meissner A Wernig M Plath K Jaenisch R Wagschal A Feil R Schreiber SL Lander ES A bivalent chromatin structure marks key developmental genes in embryonic stem cells Cell 2006 125 315 326 10.1016/j.cell.2006.02.041 16630819

[B28] McVicker G van de Geijn B Degner JF Cain CE Banovich NE Raj A Lewellen N Myrthil M Gilad Y Pritchard JK Identification of genetic variants that affect histone modifications in human cells Science 2013 342 747 749 10.1126/science.1242429 24136359 PMC3947669

[B29] Kilpinen H Waszak SM Gschwind AR Raghav SK Witwicki RM Orioli A Migliavacca E Wiederkehr M Gutierrez-Arcelus M Panousis NI Yurovsky A Lappalainen T Romano-Palumbo L Planchon A Bielser D Bryois J Padioleau I Udin G Thurnheer S Hacker D Core LJ Lis JT Hernandez N Reymond A Deplancke B Dermitzakis ET Coordinated effects of sequence variation on DNA binding, chromatin structure, and transcription Science 2013 342 744 747 10.1126/science.1242463 24136355 PMC5502466

[B30] Kasowski M Kyriazopoulou-Panagiotopoulou S Grubert F Zaugg JB Kundaje A Liu Y Boyle AP Zhang QC Zakharia F Spacek DV Li J Xie D Olarerin-George A Steinmetz LM Hogenesch JB Kellis M Batzoglou S Snyder M Extensive variation in chromatin states across humans Science 2013 342 750 752 10.1126/science.1242510 24136358 PMC4075767

[B31] Jeon JP Nam HY Shim SM Han BG Sustained viral activity of epstein-Barr virus contributes to cellular immortalization of lymphoblastoid cell lines Mol Cells 2009 27 143 148 10.1007/s10059-009-0018-y 19277495

[B32] Rashid NU Giresi PG Ibrahim JG Sun W Lieb JD ZINBA integrates local covariates with DNA-seq data to identify broad and narrow regions of enrichment, even within amplified genomic regions Genome Biol 2011 12 R67 10.1186/gb-2011-12-7-r67 21787385 PMC3218829

[B33] Boyle AP Guinney J Crawford GE Furey TS F-Seq: a feature density estimator for high-throughput sequence tags Bioinformatics 2008 24 2537 2538 10.1093/bioinformatics/btn480 18784119 PMC2732284

[B34] UCSC Genome Browser [http://genome.ucsc.edu]

[B35] Quinlan AR Hall IM BEDTools: a flexible suite of utilities for comparing genomic features Bioinformatics 2010 26 841 842 10.1093/bioinformatics/btq033 20110278 PMC2832824

[B36] Choi JK Contrasting chromatin organization of CpG islands and exons in the human genome Genome Biol 2010 11 R70 10.1186/gb-2010-11-7-r70 20602769 PMC2926781

[B37] Choi JK Bae J-B Lyu J Kim T-Y Kim Y-J Nucleosome deposition and DNA methylation at coding region boundaries Genome Biol 2009 10 R89 10.1186/gb-2009-10-9-r89 19723310 PMC2768978

[B38] Koboldt DC Chen K Wylie T Larson DE McLellan MD Mardis ER Weinstock GM Wilson RK Ding L VarScan: variant detection in massively parallel sequencing of individual and pooled samples Bioinformatics 2009 25 2283 2285 10.1093/bioinformatics/btp373 19542151 PMC2734323

[B39] Koboldt DC Zhang Q Larson DE Shen D McLellan MD Lin L Miller CA Mardis ER Ding L Wilson RK VarScan 2: somatic mutation and copy number alteration discovery in cancer by exome sequencing Genome Res 2012 22 568 576 10.1101/gr.129684.111 22300766 PMC3290792

[B40] Anthony AT Gayton BC McVicker BC An esoteric technique useful in the identification of unidentified remains from the examination of faded, illegible hospital identification wristbands J Forensic Sci 2003 48 817 820 12877299

[B41] Bennett CL Tosteson TD Schmitt B Weinberg PD Ernstoff MS Ross SD Maximum androgen-blockade with medical or surgical castration in advanced prostate cancer: A meta-analysis of nine published randomized controlled trials and 4128 patients using flutamide Prostate Cancer Prostatic Dis 1999 2 4 8 10.1038/sj.pcan.4500265 12496859

[B42] Wingender E Chen X Hehl R Karas H Liebich I Matys V Meinhardt T Pruss M Reuter I Schacherer F TRANSFAC: an integrated system for gene expression regulation Nucleic Acids Res 2000 28 316 319 10.1093/nar/28.1.316 10592259 PMC102445

[B43] Matys V Kel-Margoulis OV Fricke E Liebich I Land S Barre-Dirrie A Reuter I Chekmenev D Krull M Hornischer K Voss N Stegmaier P Lewicki-Potapov B Saxel H Kel AE Wingender E TRANSFAC and its module TRANSCompel: transcriptional gene regulation in eukaryotes Nucleic Acids Res 2006 34 D108 D110 10.1093/nar/gkj143 16381825 PMC1347505

[B44] Heinz S Benner C Spann N Bertolino E Lin YC Laslo P Cheng JX Murre C Singh H Glass CK Simple combinations of lineage-determining transcription factors prime cis-regulatory elements required for macrophage and B cell identities Mol Cell 2010 38 576 589 10.1016/j.molcel.2010.05.004 20513432 PMC2898526

[B45] Utley JR Marshall WG Boatman GB Dickerson G Ernst GB Daughtery ME Trapping, nontrapping, and release of nine and fifteen micron spheres in dog kidneys Surgery 1980 87 222 229 7355394

[B46] DNase I hypersensitive data in 70 Yoruba lymphoblastoid cell lines [http://eqtl.uchicago.edu/dsQTL_data/NORMALIZED_DATA]

[B47] Genotype data in 70 Yoruba lymphoblastoid cell lines [http://eqtl.uchicago.edu/dsQTL_data/GENOTYPES]

