# Supplementary material for: Genetic factors underlying discordance in chromatin accessibility between monozygotic twins
Source: Genome Biol. 2014 May 29;15(5):R72. doi: 10.1186/gb-2014-15-5-r72 (PMC4072931; doi:10.1186/gb-2014-15-5-r72)
Supplement: Additional file 1 — Information of the MZ twin FAIRE-seq data. [file gb-2014-15-5-r72-S1.pdf]

Table S1 Sequencing information

Sample ID	Total number of reads	Number of peak reads	Open chromatin coverage	% of >= Q30 Bases (PF)	Mean Quality Score (PF)
NIH12A5703019	141832782	64970962	61.69628367	87.72	34.83
NIH12A5320954	198584111	56924525	54.05540466	92.78	36.49
NIH12A5128512	201913217	60643088	57.58654397	91.46	36.01
NIH12A5190807	199052489	68430797	64.98173544	92.26	36.29
NIH12A5420776	198934534	51723593	49.11661099	92.63	36.41
NIH12A5369766	142691817	53489361	50.79338043	96.26	37.81
NIH12A5255708	180252172	51443840	48.85095815	95.11	37.36
NIH12A5281336	196746099	62735528	59.57352043	90.88	35.84
NIH12A5022000	201115687	71233687	67.64335366	91.57	36.04
NIH12A5647305	186065202	67696457	64.28440777	94.32	37.05
NIH12A5911884	202602432	66312942	62.97062495	92.15	36.26
NIH12A5296286	198369464	70476637	66.92446063	92.19	36.25
NIH12A5606673	201384187	66250251	62.91109371	90.76	35.73
NIH12A5967494	201973989	66181903	62.8461906	90.64	35.73
NIH12A5397033	169430551	63753809	60.54047784	95.2	37.39
NIH12A5615422	179770617	65875965	62.55567255	94.49	37.09
NIH12A5260808	164170699	69132412	65.64798751	94.07	37.01
NIH12A5870769	192721306	68842578	65.37276177	94.76	37.29
NIH12A5631255	162133373	64086180	60.85609662	94.75	37.29
NIH12A5723346	163365739	60197083	57.1630186	95.56	37.69
NIH12A5993250	191139867	71904968	68.28080063	94.88	37.32
NIH12A5416061	187386015	74708683	70.94320228	94.61	37.24
NIH12A5363794	187462893	70803238	67.23460023	94.63	37.23
NIH12A5503899	180134673	72355554	68.70867611	91.62	36.21
NIH12A5570609	149956598	64038095	60.81043521	94.9	37.35
NIH12A5973671	191521733	72348158	68.70165289	92.72	36.55
NIH12A5668468	197807135	69615783	66.10699555	92.91	36.53
NIH12A5319509	194033485	62344126	59.20184594	92.21	36.29
NIH12A5556273	187603249	54757546	51.99764614	87.21	34.67
NIH12A5868887	183163053	48627869	46.17691824	88.92	35.24
NIH12A5727187	203387098	63299518	60.1090841	91.11	35.94
NIH12A5746149	158907375	65173985	61.88907387	86.41	34.43
NIH12A5668564	180094853	65051333	61.7726038	86.87	34.51
NIH12A5599414	196061377	61024597	57.94882408	89.75	35.47
NIH12A5754559	199781473	46200605	43.87199364	89.3	35.29
NIH12A5604915	198910353	64949773	61.67616264	90.17	35.6
NIH12A5785267	195057920	38940993	36.97828194	88.65	35.12
NIH12A5198460	200025772	57382392	54.49019417	90.25	35.62
NIH12A5715967	198257581	43157992	40.98273498	90.58	35.72
NIH12A5826480	161601592	53420838	50.72831114	87.7	34.84
NIH12A5301688	159439759	45403331	43.11490399	88.03	34.93
NIH12A5032877	188779281	34378772	32.64600684	90.33	35.69
NIH12A5027049	203877062	47010045	44.6406361	91.03	35.91
NIH12A5025633	192664233	70502092	66.94863265	94.43	37.13
NIH12A5436255	204221573	39396891	37.41120168	92.39	36.37
NIH12A5972531	194967933	40892794	38.83170791	94.06	36.99
NIH12A5676841	204026983	55535469	52.73636012	92.63	36.43
NIH12A5053877	208573702	71712871	68.09838574	91.31	35.98
NIH12A5263748	199269220	69015523	65.53698997	93.9	36.93
NIH12A5355009	200063583	53527926	50.83000167	93.46	36.78
NIH12A5793958	192510417	49487373	46.99310136	94.36	37.11
NIH12A5140093	198482334	60456215	57.40908978	94.01	36.95
NIH12A5049732	211914660	65095100	61.81416485	91.07	35.9
NIH12A5734960	200160981	68339931	64.89544928	93.38	36.74
NIH12A5117243	205743527	65364518	62.07000359	91.48	36.05
NIH12A5885614	192740655	76309180	72.46303073	91.92	36.3

NIH12A5144119	198091789	65635875	62.32768361	90.22	35.62
NIH12A5400104	202309598	57549543	54.64892039	92.26	36.3
NIH12A5146351	204178042	72913230	69.23824403	91.6	36.1
NIH12A5467537	204697006	68178497	64.74215191	89.92	35.53
NIH12A5089950	189601159	72057602	68.42574155	93.18	36.68
NIH12A5937541	186858288	51342035	48.75428435	89.87	35.54
NIH12A5243416	157710335	57156650	54.27583006	92.26	36.33
NIH12A5413827	190934486	72257123	68.61520625	92.34	36.39
NIH12A5055252	160076285	67976806	64.55062657	91.47	36.04
NIH12A5716614	193166891	70405686	66.85708572	93.35	36.72
NIH12A5424517	188777292	62450304	59.30267234	88.53	35.06
NIH12A5731088	203261220	66305170	62.96324468	92.2	36
NIH12A5962416	171488918	74106950	70.37179793	94.51	37
NIH12A5072062	193178002	64485471	61.23526248	92.62	36
NIH12A5054682	203097169	55678431	52.87211652	90.19	36
NIH12A5023630	198471820	64869583	61.6000144	90.52	36
