# Supplementary material for: Genetic factors underlying discordance in chromatin accessibility between monozygotic twins
Source: Genome Biol. 2014 May 29;15(5):R72. doi: 10.1186/gb-2014-15-5-r72 (PMC4072931; doi:10.1186/gb-2014-15-5-r72)
Supplement: Additional file 2 — Clinical information of the MZ twins used in this work. [file gb-2014-15-5-r72-S2.pdf]

Table S2 Clinical information of MZ twins

MZ Twin ID		Sex	Age	Discordant for
Control	Case			
NIH12A5615422	NIH12A5397033	M	46	Allergic rhinitis
NIH12A5868887	NIH12A5556273	M	36	Allergic rhinitis
NIH12A5301688	NIH12A5032877	M	31	Allergic rhinitis
NIH12A5972531	NIH12A5436255	M	31	Allergic rhinitis
NIH12A5369766	NIH12A5420776	F	41	Allergic rhinitis
NIH12A5281336	NIH12A5255708	F	44	Allergic rhinitis
NIH12A5647305	NIH12A5022000	F	44	Allergic rhinitis
NIH12A5870769	NIH12A5260808	F	46	Allergic rhinitis
NIH12A5668564	NIH12A5599414	F	31	Allergic rhinitis
NIH12A5604915	NIH12A5754559	F	31	Allergic rhinitis
NIH12A5826480	NIH12A5715967	F	49	Allergic rhinitis
NIH12A5027049	NIH12A5025633	F	32	Allergic rhinitis
NIH12A5400104	NIH12A5144119	F	35	Allergic rhinitis
NIH12A5937541	NIH12A5089950	F	35	Allergic rhinitis
NIH12A5198460	NIH12A5785267	F	33	Allergic rhinitis
NIH12A5190807	NIH12A5128512	F	41	Allergic rhinitis, Allergic conjunctivitis, Arthritis
NIH12A5413827	NIH12A5243416	M	34	Allergic rhinitis, Allergic conjunctivitis
NIH12A5072062	NIH12A5962416	M	34	Allergic rhinitis, Allergic conjunctivitis
NIH12A5355009	NIH12A5263748	M	49	Allergic rhinitis, Arthritis
NIH12A5023630	NIH12A5054682	F	35	Allergic rhinitis, Atopic dermatitis
NIH12A5140093	NIH12A5793958	M	33	Atopic dermatitis
NIH12A5049732	NIH12A5734960	M	47	Atopic dermatitis
NIH12A5117243	NIH12A5885614	M	31	Atopic dermatitis
NIH12A5296286	NIH12A5911884	F	44	Atopic dermatitis
NIH12A5723346	NIH12A5631255	F	47	Atopic dermatitis
NIH12A5973671	NIH12A5570609	F	32	Atopic dermatitis
NIH12A5668468	NIH12A5319509	F	41	Atopic dermatitis, Allergic conjunctivitis
NIH12A5320954	NIH12A5703019	F	41	Asthma, Allergic rhinitis
NIH12A5967494	NIH12A5606673	F	45	Asthma, Allergic rhinitis
NIH12A5746149	NIH12A5727187	F	30	Asthma, Allergic rhinitis
NIH12A5503899	NIH12A5363794	F	50	Asthma, Arthritis
NIH12A5053877	NIH12A5676841	F	32	Allergic conjunctivitis
NIH12A5467537	NIH12A5146351	M	55	Arthritis
NIH12A5416061	NIH12A5993250	F	48	Arthritis
NIH12A5716614	NIH12A5055252	F	45	Arthritis
NIH12A5731088	NIH12A5424517	F	54	Arthritis
