# Supplementary material for: Genetic factors underlying discordance in chromatin accessibility between monozygotic twins
Source: Genome Biol. 2014 May 29;15(5):R72. doi: 10.1186/gb-2014-15-5-r72 (PMC4072931; doi:10.1186/gb-2014-15-5-r72)
Supplement: Additional file 3 — Density plot for genome-wide correlation coefficients of chromatin accessibility between twin siblings and between unrelated individuals. [file gb-2014-15-5-r72-S3.pdf]

Figure S1

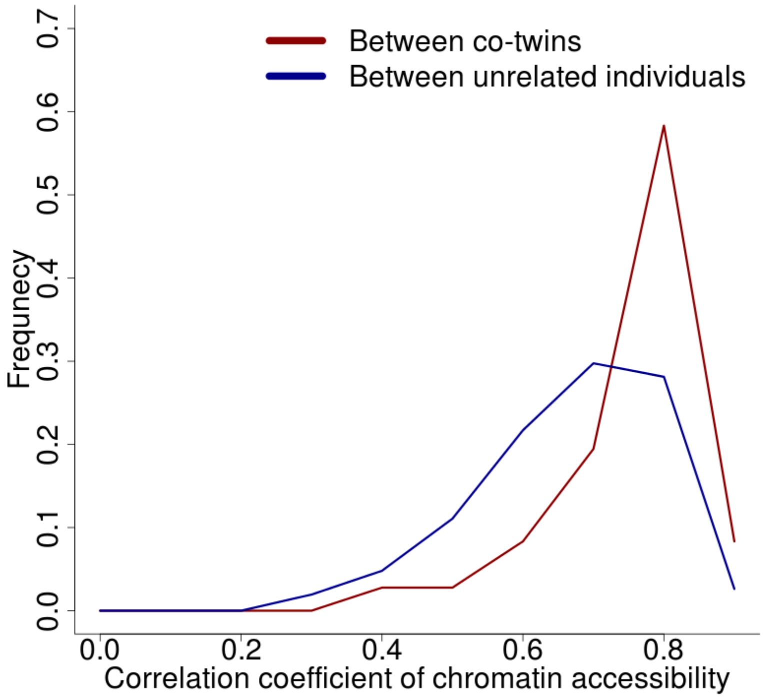

Density plot for genome-wide correlation coefficients of chromatin accessibility between twin siblings and between unrelated individuals.
