# Supplementary material for: Genetic factors underlying discordance in chromatin accessibility between monozygotic twins
Source: Genome Biol. 2014 May 29;15(5):R72. doi: 10.1186/gb-2014-15-5-r72 (PMC4072931; doi:10.1186/gb-2014-15-5-r72)
Supplement: Additional file 4 — The number of open chromatin regions we identified that overlap with peaks identified from the public FAIRE-seq and DNase-seq data for the GM12878 lymphoblastoid cells. Our FAIRE regions were extended 10 to 200 bp before overlapping. Approximately 83% of the FAIRE regions that we found to overlap with the public FAIRE data were confirmed by the public DNase I data. [file gb-2014-15-5-r72-S4.pdf]

Figure S2

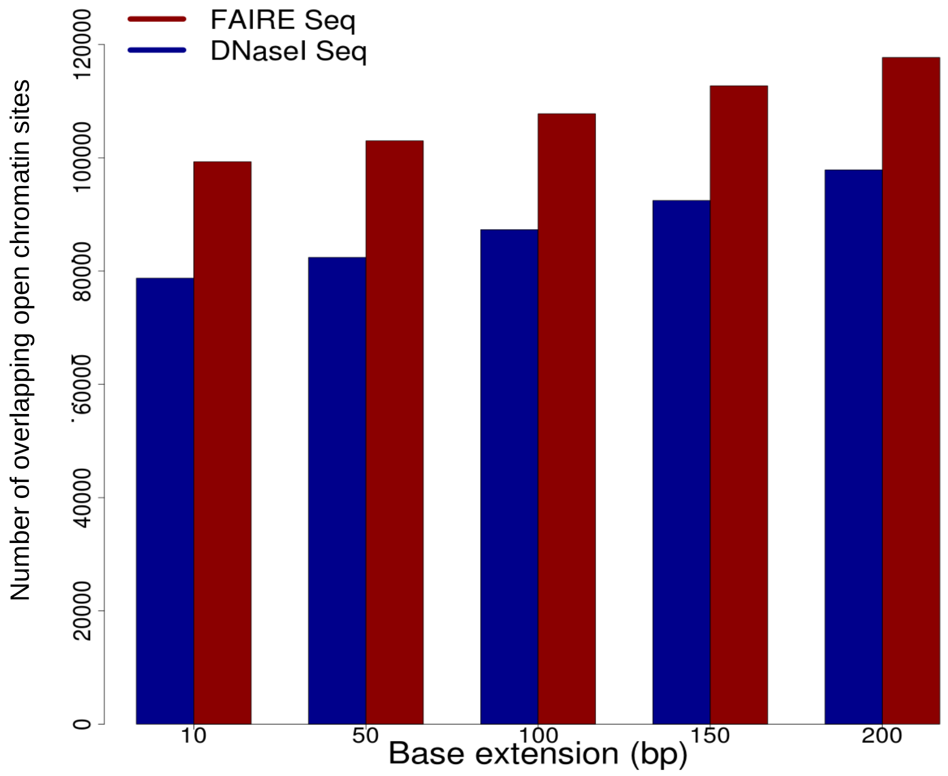

The number of open chromatin regions we identified that overlap with peaks identified from the public FAIRE-seq and DNase-seq data for the GM12878 lymphoblastoid cells. Our FAIRE regions were extended 10 ~ 200 bp before overlapping. Approximately 83% of the FAIRE regions that we found to overlap with the public FAIRE data were confirmed by the public DNase I data.
