# Supplementary material for: Genetic factors underlying discordance in chromatin accessibility between monozygotic twins
Source: Genome Biol. 2014 May 29;15(5):R72. doi: 10.1186/gb-2014-15-5-r72 (PMC4072931; doi:10.1186/gb-2014-15-5-r72)
Supplement: Additional file 7 — Within-pair differences in chromatin accessibility as a function of the distance between the mutation and the center of the chromatin region. [file gb-2014-15-5-r72-S7.pdf]

Figure S3

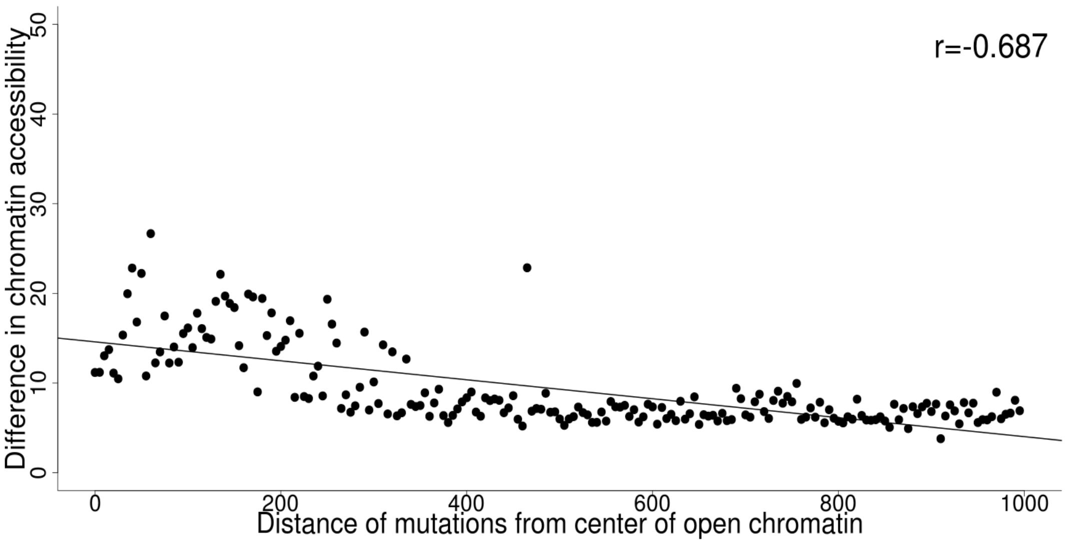

Within-pair differences in chromatin accessibility as a function of the distance between the mutation and the center of the chromatin region.
