# Supplementary material for: Genetic factors underlying discordance in chromatin accessibility between monozygotic twins
Source: Genome Biol. 2014 May 29;15(5):R72. doi: 10.1186/gb-2014-15-5-r72 (PMC4072931; doi:10.1186/gb-2014-15-5-r72)
Supplement: Additional file 8 — Within-pair differences in chromatin accessibility according to the number of mutations per base pair as an estimate of the density of mutations relative to the size of the open chromatin region. [file gb-2014-15-5-r72-S8.pdf]

Figure S4

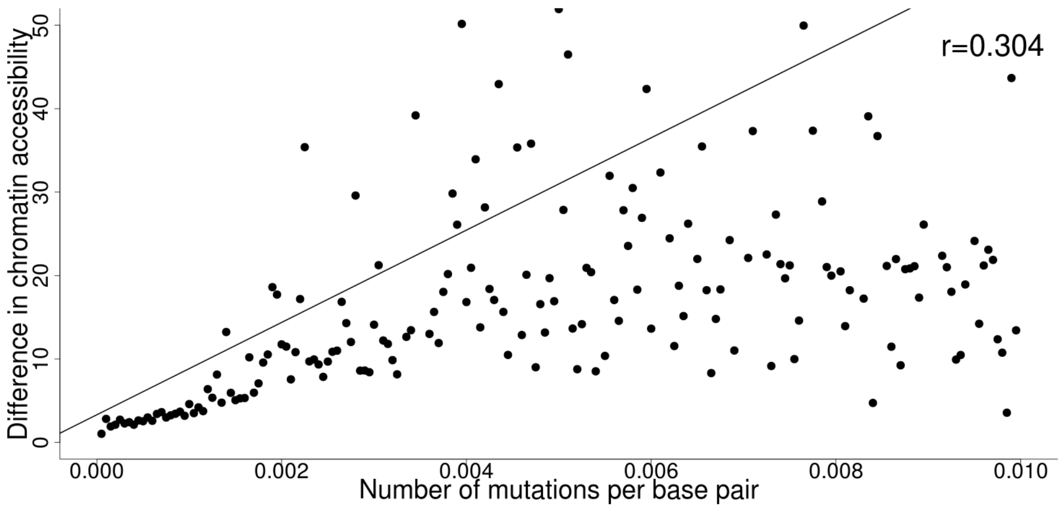


Within-pair differences in chromatin accessibility according to the number of mutations per base pair as an estimate of the density of mutations relative to the size of the open chromatin region.
