# Supplementary material for: Genetic factors underlying discordance in chromatin accessibility between monozygotic twins
Source: Genome Biol. 2014 May 29;15(5):R72. doi: 10.1186/gb-2014-15-5-r72 (PMC4072931; doi:10.1186/gb-2014-15-5-r72)
Supplement: Additional file 9 — Observed-to-expected ratios of the substitution frequency of TFBS mutations and polymorphisms that were associated with chromatin discordance and inter-individual variation, respectively. [file gb-2014-15-5-r72-S9.pdf]

Figure S5

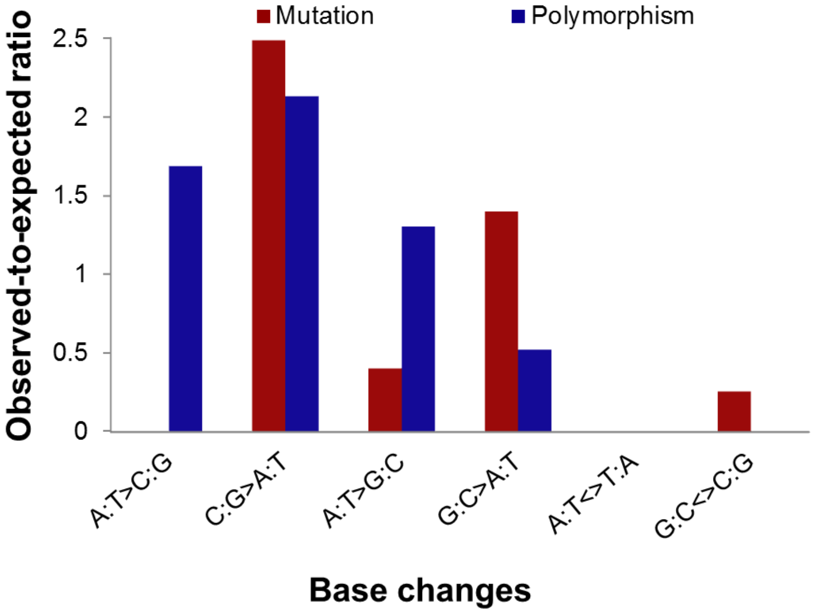

Observed-to-expected ratios were defined as the ratio of the substitution frequency of mutations and polymorphisms in TFBSs to the overall substitution frequency in open chromatin, in the case of association with chromatin discordance and inter-individual variation
