# Supplementary material for: Genetic factors underlying discordance in chromatin accessibility between monozygotic twins
Source: Genome Biol. 2014 May 29;15(5):R72. doi: 10.1186/gb-2014-15-5-r72 (PMC4072931; doi:10.1186/gb-2014-15-5-r72)
Supplement: Additional file 10 — The relative enrichment of dinucleotides in TFBSs. The ratio of the number of the specified dinucleotides in TFBSs to the number in the surrounding chromatin regions was divided by the ratio for all the different dinucleotides. [file gb-2014-15-5-r72-S10.pdf]

Figure S6

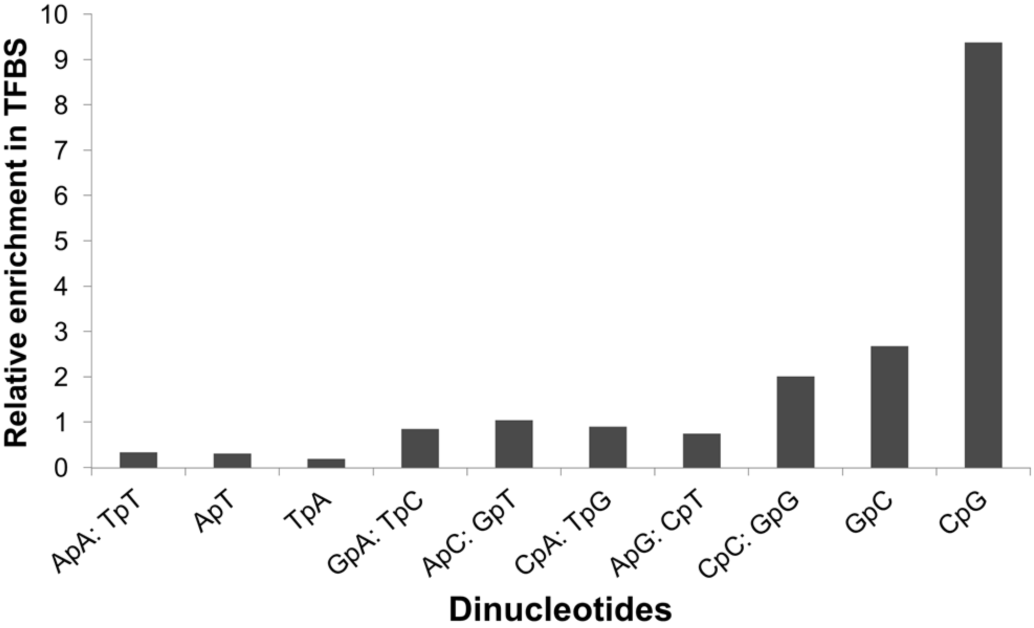


The relative enrichment of dinucleotides in TFBSs. The ratio of the number of the specified dinucleotides in TFBSs to the number in the surrounding chromatin regions was divided by the ratio for all the different dinucleotides
