# Supplementary material for: Genetic factors underlying discordance in chromatin accessibility between monozygotic twins
Source: Genome Biol. 2014 May 29;15(5):R72. doi: 10.1186/gb-2014-15-5-r72 (PMC4072931; doi:10.1186/gb-2014-15-5-r72)
Supplement: Additional file 11 — The relative enrichment of dinucleotides in TFBSs. The ratio of the number of each dinucleotide in TFBSs to the number in the whole genome was divided by the ratio of the number of all the different dinucleotides in TFBSs to the number in the whole genome. [file gb-2014-15-5-r72-S11.pdf]

Figure S7

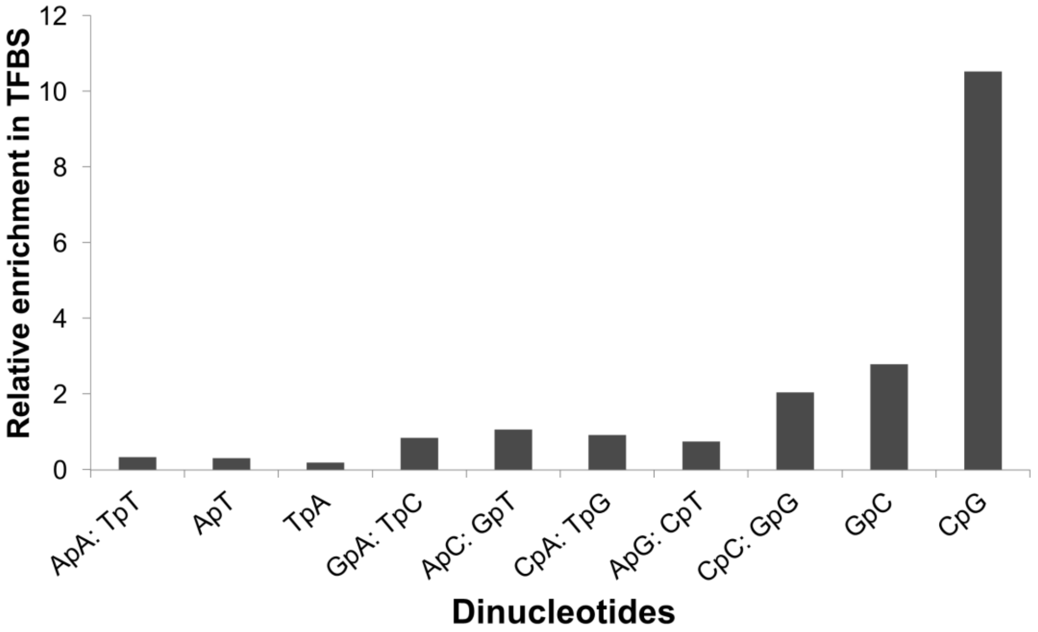

The relative enrichment of dinucleotides in TFBSs. The ratio of the number of each dinucleotide in TFBSs to the number in the whole genome was divided by the ratio of the number of all the different dinucleotides in TFBSs to the number in the whole genome.
