# Supplementary material for: Genetic factors underlying discordance in chromatin accessibility between monozygotic twins
Source: Genome Biol. 2014 May 29;15(5):R72. doi: 10.1186/gb-2014-15-5-r72 (PMC4072931; doi:10.1186/gb-2014-15-5-r72)
Supplement: Additional file 12 — Differential methylation levels between B lymphocytes and other cell types (embryonic stem cells (ESC), hematopoietic progenitor cells (HPC), and neutrophils) for each TFBS CpG. The average of cytosine methylations in TFBSs was obtained and plotted. [file gb-2014-15-5-r72-S12.pdf]

Figure S8

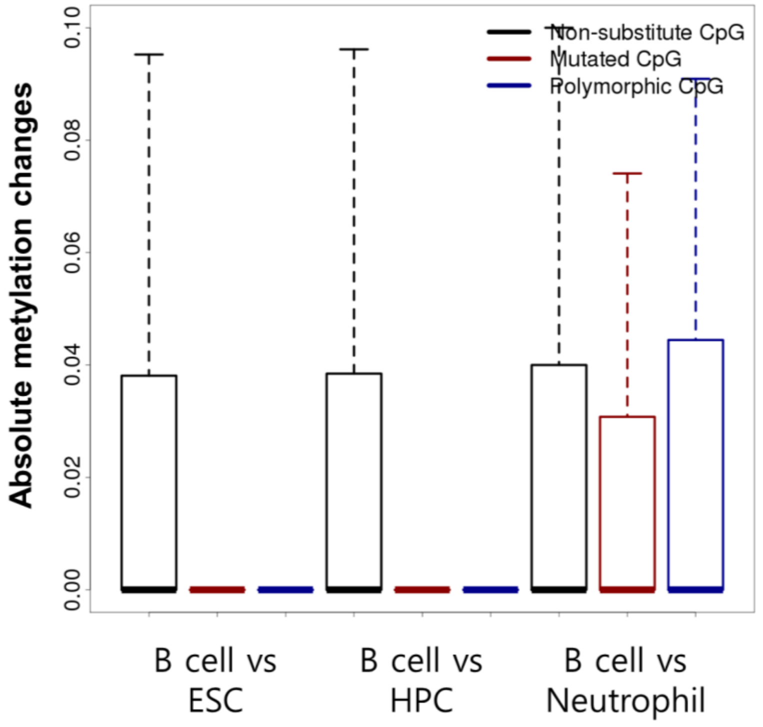

For each TFBS CpG, differential methylation levels between B lymphocytes and other cell types (ESC: embryonic stem cell, HPC: hematopoietic progenitor cell, and Neutrophil) were calculated. The average of cytosine methylations in TFBSs was obtained and plotted.
