# Supplementary material for: Genetic factors underlying discordance in chromatin accessibility between monozygotic twins
Source: Genome Biol. 2014 May 29;15(5):R72. doi: 10.1186/gb-2014-15-5-r72 (PMC4072931; doi:10.1186/gb-2014-15-5-r72)
Supplement: Additional file 13 — The frequency of different types of polymorphisms arising in TFBSs as identified in this work (dark blue) and in the previous work by Degner et al. [13] (sky blue). Only changes from the reference homozygote were considered. [file gb-2014-15-5-r72-S13.pdf]

Figure S9

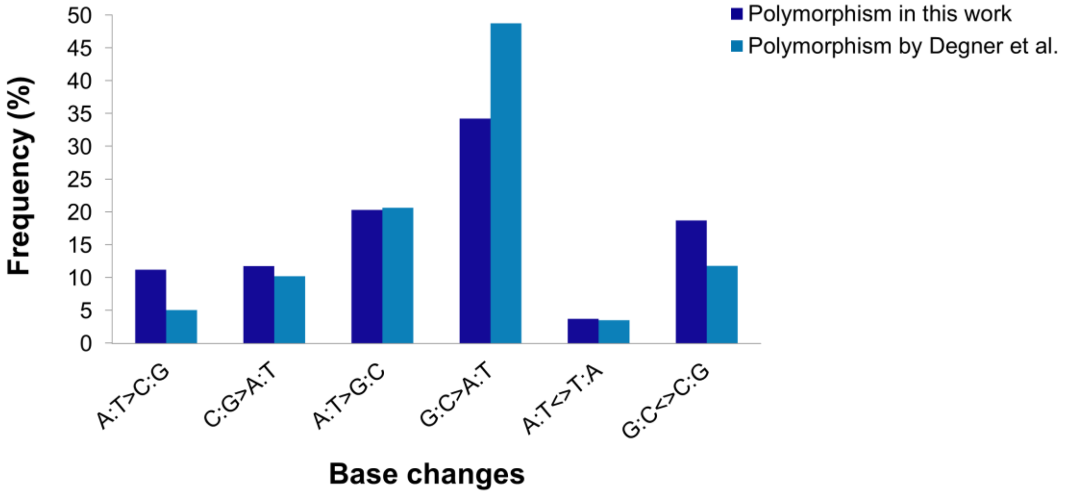

The frequency of different types of polymorphisms arising in TFBSs as identified in this work (dark blue) and in the previous work by Degner et al. [13] (sky blue). Only changes from the reference homozygote were considered.
