# Supplementary material for: Genetic factors underlying discordance in chromatin accessibility between monozygotic twins
Source: Genome Biol. 2014 May 29;15(5):R72. doi: 10.1186/gb-2014-15-5-r72 (PMC4072931; doi:10.1186/gb-2014-15-5-r72)
Supplement: Additional file 14 — The frequency of TFBS dinucleotides containing polymorphisms identified in this work (dark blue) and in the previous work by Degner et al . [13] (sky blue). [file gb-2014-15-5-r72-S14.pdf]

Figure S10

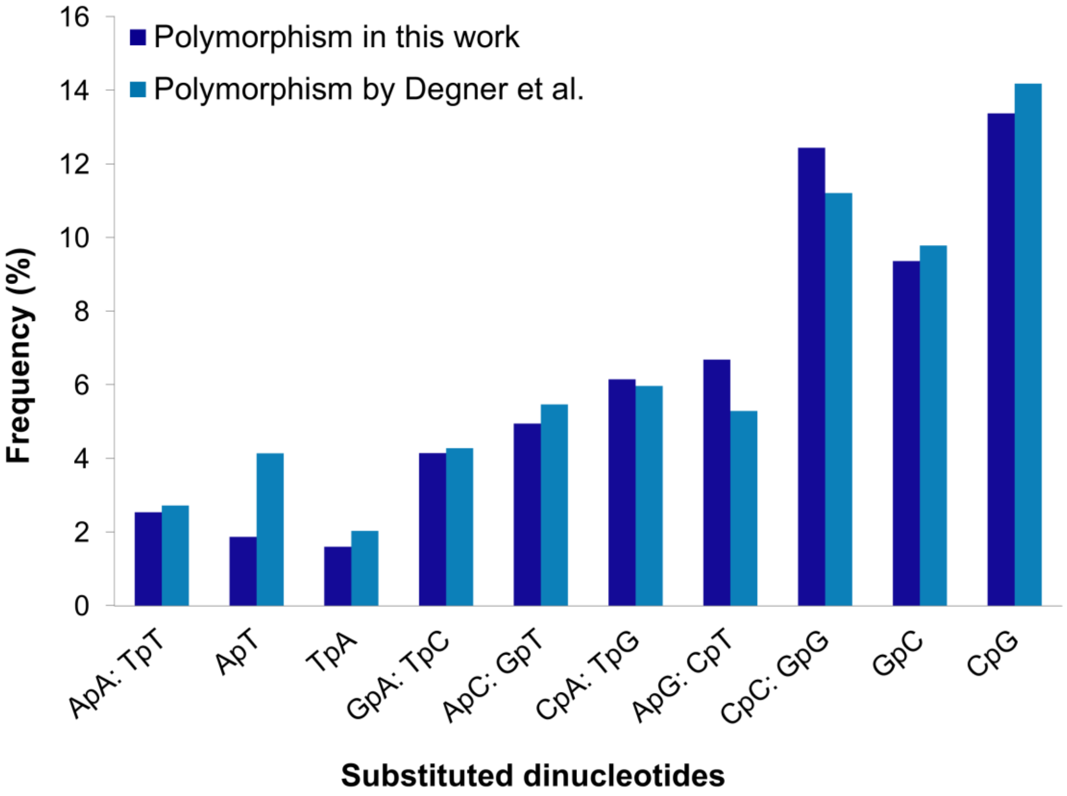

The frequency of TFBS dinucleotides containing polymorphisms identified in this work (dark blue) and in the previous work by Degner et al. [13] (sky blue).
