# Supplementary material for: Genetic factors underlying discordance in chromatin accessibility between monozygotic twins
Source: Genome Biol. 2014 May 29;15(5):R72. doi: 10.1186/gb-2014-15-5-r72 (PMC4072931; doi:10.1186/gb-2014-15-5-r72)
Supplement: Additional file 15 — Left: for the TFBS polymorphisms identified by Degner et al. [13], observed-to-expected ratios were obtained as the ratio of the relative enrichment of polymorphic dinucleotides in TFBSs to the relative enrichment of all dinucleotides in TFBSs. Right: for each TFBS CpG, differential methylation levels between B lymphocytes and other cell types (embryonic stem cells (ESC), hematopoietic progenitor cells (HPC), and neutrophils) were calculated. [file gb-2014-15-5-r72-S15.pdf]

Figure S11

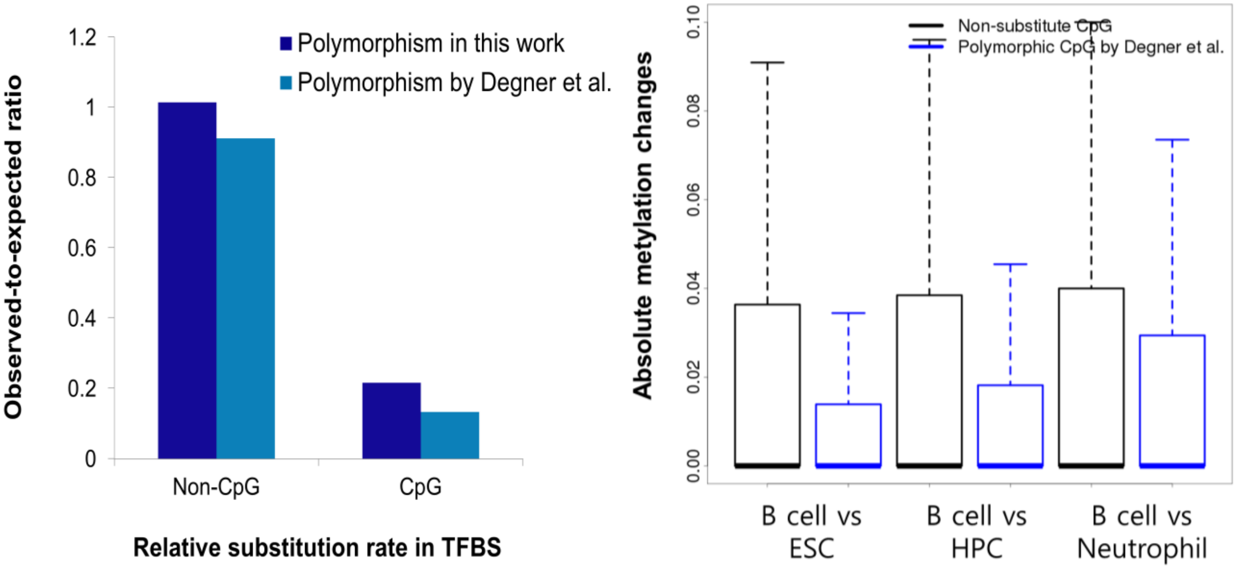

[Left] For the TFBS polymorphisms identified by Degner et al. [13], observed-to-expected ratios were obtained as the ratio of the relative enrichment of polymorphic dinucleotides in TFBSs to the relative enrichment of all dinucleotides in TFBSs.

[Right] For each TFBS CpG, differential methylation levels between B lymphocytes and other cell types (ESC: embryonic stem cell, HPC: hematopoietic progenitor cell, and neutrophil) were calculated.
